# Balancing adipocyte production and lipid metabolism to treat obesity-induced diabetes with a novel proteoglycan from *Ganoderma lucidum*

**DOI:** 10.1186/s12944-023-01880-6

**Published:** 2023-08-08

**Authors:** YingXin Wang, Fanzhen Yu, Xinru Zheng, Jiaqi Li, Zeng Zhang, Qianqian Zhang, Jieying Chen, Yanming He, Hongjie Yang, Ping Zhou

**Affiliations:** 1 State Key Laboratory of Molecular Engineering of Polymers, Department of Macromolecular Science, Fudan University, Shanghai, 200433 China; 2 Yueyang Hospital of Integrated Traditional Chinese and Western Medicine, Shanghai University of Traditional Chinese Medicine, Shanghai, 200437 China

**Keywords:** Metabolic disorder, Obesity, Diabetes, *Ganoderma lucidum*, Adipocytes, Lipid metabolism, 3T3-L1, AMPKα signalling pathway

## Abstract

Obesity is often accompanied by metabolic disorder and insulin resistance, resulting in type 2 diabetes. Based on previous findings, *FYGL*, a natural hyperbranched proteoglycan extracted from the *G. lucidum* fruiting body, can decrease blood glucose and reduce body weight in diabetic mice. In this article, the underlying mechanism of *FYGL* in ameliorating obesity-induced diabetes was further investigated both in vivo and in vitro. *FYGL* upregulated expression of metabolic genes related to fatty acid biosynthesis, fatty acid β-oxidation and thermogenesis; downregulated the expression of insulin resistance-related genes; and significantly increased the number of beige adipocytes in *db/db* mice. In addition, *FYGL* inhibited preadipocyte differentiation of 3T3-L1 cells by increasing the expression of FABP-4. *FYGL* not only promoted fatty acid synthesis but also more significantly promoted triglyceride degradation and metabolism by activating the AMPK signalling pathway, therefore preventing fat accumulation, balancing adipocyte production and lipid metabolism, and regulating metabolic disorders and unhealthy obesity. *FYGL* could be used as a promising pharmacological agent for the treatment of metabolic disorder-related obesity.

**Supplementary Information:**

The online version contains supplementary material available at 10.1186/s12944-023-01880-6.

## Introduction

Type 2 diabetes mellitus (T2DM) is a chronic degenerative disease, and 60% of T2DM patients are obese as a result of metabolic disorder and insulin resistance as well as impaired energy homoeostasis [1–6]. Adipose tissues play an important role in surplus energy storage and energy metabolism [4]. Adipose tissue comprises white adipose tissue (WAT), brown adipose tissue and beige adipose tissue. White adipose tissue mainly functions to store fat in the form of lipid droplets and secrete adipokines to regulate the metabolism of tissues such as muscle and liver tissues [7]. Brown and beige adipose tissues mainly act to dissipate excess energy through thermogenesis to maintain a stable body weight, and secrete many batokines to affect the physiology of a variety of organ systems and tissues, such as liver, heart and muscle [8, 9]. Accumulating evidence has suggested that a high ratio of white to beige adipocytes is associated with insulin resistance [3, 5]. The beige adipose tissue play more roles for controlling the metabolically unhealthy obesity than other adipose tissue [5].

Adipocytes are differentiated from preadipocytes, therefore, many studies have focused on inhibiting the differentiation of preadipocytes to treat obesity in addition to regulating lipid metabolism [10, 11]. Mesenchymal stem cells (MSCs) undergo a two-step process to differentiate into adipocytes: MSCs first differentiate into preadipocytes, and then preadipocytes continue to differentiate into mature adipocytes [12, 13]. During adipogenesis, the peroxisome proliferator-activated receptor gamma (PPARγ) and CCAAT/enhancer-binding protein α (C/EBPα) are marker proteins for preadipocytes differentiating into mature adipocytes [14, 15]. Subsequently, fatty acids are synthesized in conjunction with the expressions of acetyl-CoA carboxylase (ACCα) and fatty acid synthase (FAS). Moreover, mature adipocytes further synthesize triglycerides, which aggregate to form lipid droplets [16]. In addition, the triglycerides in lipid droplets are degraded in conjunction with the expressions of adipose triglyceride lipase (ATGL), hormone-sensitive lipase (HSL) [17, 18], which are regulated by the AMP-activated protein kinase α (AMPK α) signalling pathway; thermogenesis in beige adipose tissue is also regulated by this pathway [19, 20]. Based on these results, finding an effective agent to regulate metabolic disorders and control obesity-induced diabetes is very important.

Some anti-obesity drugs, such as orlistat and liraglutide, have been applied clinically in recent years [21, 22]. Orlistat controls body weight by inhibiting pancreatic lipases but has side effects, such as faecal incontinence and flatulence [23]. Liraglutide controls body weight by suppressing gastric emptying and food intake, increasing satiety, and limiting nutrient absorption by increasing pancreatic β cell proliferation, regenerating β cells, and alleviating insulin resistance, but also has side effects such as nausea, vomiting, and diarrhoea [24]. Metformin, a first-line therapeutic agent for diabetes, is an AMPK activator capable of increasing insulin sensitivity and decreasing body weight, but it also has side effects such as abdominal distension, diarrhoea and gastrointestinal intolerance [25].

**Fig. 1:**
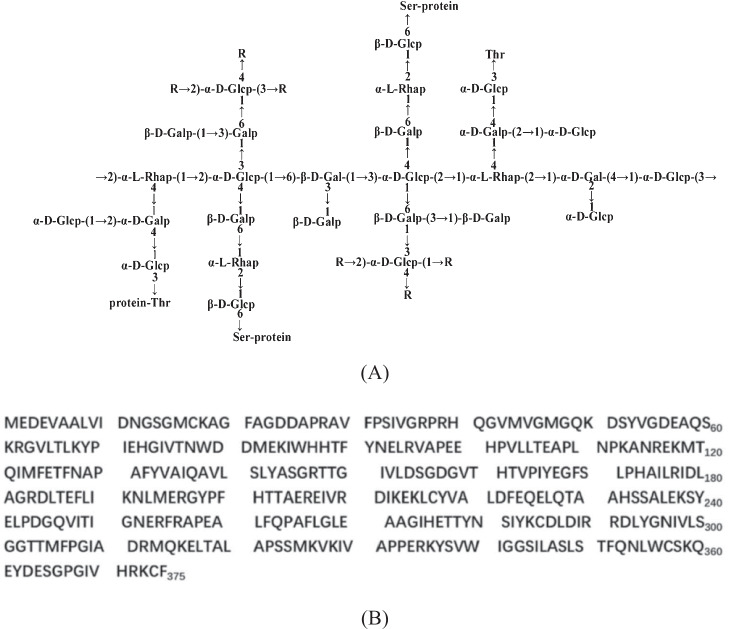
**A** The dominant polysaccharide sequence of *FYGL* characterized by chemical analysis and NMR spectroscopy [29]. Rs represents the carbohydrate residues of → 2,4)-α-L-Rhap-(1 → , → 6-β-D-Galp-1 → , Araf-(1 → or → 3,6)-β-D-Galp-(1 → . Protein moieties are covalently bonded with carbohydrate moieties by Ser and Thr residues in the -O- linkage. **B** The dominant sequence of the protein moieties of *FYGL* characterized by mass spectrometry

Previously, Teng et al. extracted a proteoglycan called *FYGL* (Fudan-Yueyang *G. lucidum*) from the fruiting body of *Ganoderma lucidum*, a traditional Chinese medicinal herb used for immunoregulation, anti-inflammation, anti-diabetes and anti-cancer [26, 27]. The dominant sequence of *FYGL* is shown in Fig. 1 [28, 29]. *FYGL* is a hyperbranched proteoglycan with a molecular weight of 2.6 × 10^5^ Da and a saccharide: protein ratio of 77:17 [28, 29]. The content of *FYGL* in *Ganoderma lucidum* is about 1% [28]. *FYGL* has been proven capable of decreasing fasting blood glucose through inhibition of the activity of protein tyrosine phosphatase 1 B (PTP1B), an insulin resistance receptor, both in vitro [30] and in vivo [31, 32], as well as reducing body weight in *ob/ob* mice [33]. However, the underlying mechanism by which *FYGL* controls body weight is unknown.

**Fig. 2:**
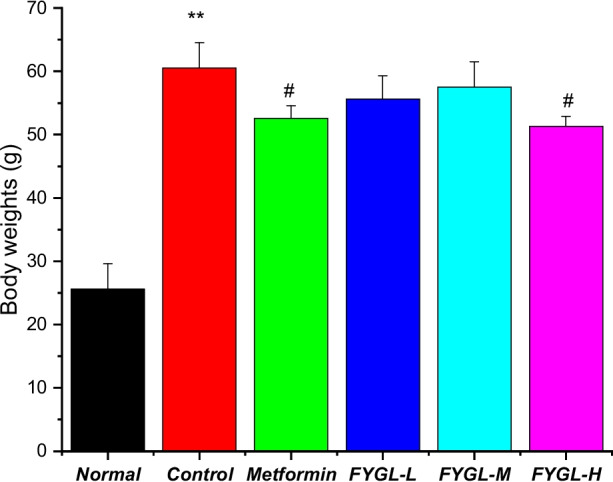
Body weights of *db/db* mice treated with *FYGL* for 8 weeks. *P < 0.05, **P < 0.01 *vs.* control group [34]

In this work, the mechanism of *FYGL* against obesity-induced diabetes was investigated both in vivo and in vitro. In in vivo studies, *db/db* mice, a specific model of obesity and diabetes resulting from receptor leptin resistance, were used in this work. Because body weight (Fig. 2) and basic metabolic data (including blood glucose, serum insulin level, glycosylated haemoglobin as well as serum triglycerides (TG), total cholesterol (TC), low density lipoprotein-cholesterol (LDL-c), high density lipoprotein-cholesterol (HDL-c) of the animals have been studied and published previously (Figure S1 and S2 in Supplementary materials) [34], this paper focused on the function and mechanism of *FYGL* controlling obesity. The beige adipose tissue from *db/db* diabetic mice was used to analyse the expression of genes related to fatty acid biosynthesis and metabolism, thermogenesis, and insulin sensitivity, which are beneficial for beige adipose tissue functions. In in vitro studies, the 3T3-L1 cell line was used to investigate the underlying mechanism by which *FYGL* alleviates obesity. 3T3-L1 cells are preadipocytes and normally differentiate into mature adipocytes [35]. The effects of *FYGL* on preadipocyte differentiation and mature adipocyte lipid metabolism were investigated in terms of gene and protein expressions in preadipocytes as well as signalling pathways of lipid metabolism in mature adipocytes.

## Materials and methods

### Materials

Fruiting bodies of *G. lucidum* grown in northeast China were purchased from Leiyunshang Pharmaceutical Co. Ltd (Shanghai, China). The preparation of *FYGL* was described in previous work [31]. Dulbecco's modified Eagle's medium (DMEM), foetal bovine serum (FBS), and penicillin/streptomycin antibiotics were purchased from Gibco Co. Ltd (USA). 3T3-L1 cells were obtained from Procell Life Science & Technology Co. Ltd (Wuhan, China). Fluorescein isothiocyanate (FITC), 4ʹ,6-diamidino-2-phenylindole (DAPI), rhodamine-labelled phalloidin and super ECL detection reagent were provided by Yeasen Co. Ltd (Shanghai, China). A cell counting kit-8 (CCK-8), a modified oil red O staining kit, a bicinchoninic acid (BCA) kit, newborn calf serum (NCS), RIPA lysis buffer, dexamethasone, 3-isobutyl-1-methylxanthine (IBMX), paraformaldehyde, Triton X-100, anti-rabbit IgG (H + L), and a horseradish peroxidase (HRP)-labelled secondary antibody were purchased from Beyotime Co. Ltd (Shanghai, China). Dimethyl sulfoxide (DMSO) was provided by Sigma–Aldrich (Taufkirchen, Germany). Triglyceride (TG) assay kits were obtained from Jiancheng Bioengineering Institute (Nanjing, China). The RNAprep pure cell kit was acquired from TIANGEN Biotech Co. Ltd (Beijing, China). The HiScript III All-in-one RT SuperMix kit (#R333) and Taq Pro Universal SYBR qPCR Master Mix kit (#Q712) were purchased from Vazyme Biotech Co. Ltd (Nanjing, China). Primary antibodies against peroxisome proliferator-activated receptor gamma (PPAR, A11183), lipoprotein lipase (LPL, A16252), and β-actin (AC026) were purchased from ABclonal Technology Co. Ltd (Wuhan, China). Primary antibodies against CCAAT/enhancer-binding protein α (C/EBPα, ab40764), fatty acid synthase (FAS, ab128870), fatty acid binding protein 4 (FABP-4, ab92501), adipose triglyceride lipase (ATGL, ab109251), AMPKα1 (ab32047), AMPKα1 (phospho T183) + AMPKα2 (phospho T172) (p-AMPKα, ab133448) were purchased from Abcam (Cambridge, MA, USA). Primary antibodies against hormone-sensitive lipase (HSL, #4107) were purchased from Cell Signaling Technology (CST, Beverly, MA, USA).

### Animal trial

All male C57BLKS/J-Diabetes Genes (BKS-DB) mice (4 weeks old) and wild-type BKS-DB (*db/m*) mice were purchased from GemPharmatech Co. Ltd, Nanjing, China. Mice were housed in the specific pathogen-free (SPF) animal experimental center of the school of pharmacy, Fudan University, at a constant temperature (22 ± 2 ℃) on a 12 h/12 h light/dark cycle and were provided standard food and water. All animal trials were conducted following protocols approved by the Fudan University Institutional Animal Care and Use Committee. Subsequent experimental procedures were performed according to the method described in previous works [36, 37]. After adaptation for 4 weeks, the mice were divided into six groups (12 mice/group): (1) *db/m* mice + saline (normal); (2) *db/db* + saline (control); (3) *db/db* + metformin (225 mg/kg/d, positive); (4–6) *db/db* + *FYGL* (225 mg/kg/d, 450 mg/kg/d, 900 mg/kg/d, as low (*FYGL*-L), middle (*FYGL*-M), high (*FYGL*-H) dose, respectively). Six groups were orally administrated with saline or the corresponding concentrations of drugs for 8 weeks. During the experiment, the body weight of all the mice was recorded weekly as a basis for gavage dose, and FBG levels of blood samples from the tail vein were examined weekly [34]. After 7 weeks of drug treatment, all the mice were sacrificed by cervical dislocation.

### Histopathological analysis of beige adipose tissue

Beige adipose tissue was extracted from the scapulae of *db/db* mice and were fixed, sectioned, and mounted. The sections were stained with haematoxylin and eosin (H&E) and observed by microscopy (NanoZoomer 2.0-HT, Japan). Adipocyte numbers are shown as ratios of the adipocyte number to the area of the selected region (a randomly selected circle with an area of 0.1 mm^2^) in the images.

### RNA sequencing (RNA-seq) analysis of beige adipose tissue

Total RNA was extracted from beige adipose tissue. RNA purity was checked using a NanoPhotometer® spectrophotometer (IMPLEN, CA, USA). RNA integrity was assessed using the RNA Nano 6000 Assay Kit for the Bioanalyzer 2100 system (Agilent Technologies, CA, USA). Sequencing libraries were generated using the NEBNext® UltraTM RNA Library Prep Kit for Illumina® (NEB, USA) following the manufacturer’s recommendations, and index codes were added to attribute sequences to each sample. Clustering of the index-coded samples was performed on a cBot Cluster Generation System using TruSeq PE Cluster Kit v3-cBot-HS (Illumina) according to the manufacturer’s instructions. After cluster generation, the library preparations were sequenced on the Illumina NovaSeq platform, and 150 bp paired-end reads were generated. Every group was analysed with three biological replicates. Differential expression analyses between two conditions or groups (two biological replicates per condition) were performed using the DESeq2 R package (1.16.1). Genes with an adjusted *P* value of < 0.05 determined by DESeq2 were considered differentially expressed. Gene Ontology (GO) enrichment analysis of the differentially expressed genes was implemented in the cluster Profiler R package, in which gene length bias was corrected. GO terms with a corrected *P* value of less than 0.05 were considered significantly enriched with differentially expressed genes. The clusterProfiler R package was used to test the statistical enrichment of the differentially expressed genes in Kyoto Encyclopedia of Genes and Genomes (KEGG) for signalling pathway analysis.

### Cell culture and treatment

3T3-L1 preadipocytes were maintained in DMEM supplemented with 10% NCS and 1% penicillin‒streptomycin (basal medium I, BMI). When the cells were confluent (Day 0), adipocyte differentiation was induced by treatment with a cocktail of 5 μg/mL insulin, 1 μM dexamethasone, and 0.5 mM isobutyl methylxanthine in DMEM supplemented with 10% FBS and 1% penicillin‒streptomycin (differentiation medium I, DMI). After 48 h (Day 2), the medium was changed to DMEM containing 10% FBS, 1% penicillin–streptomycin, and 5 μg/mL insulin for 48 h (differentiation medium II, DMII). On Day 4, insulin was removed from the medium, and the cells were maintained in DMEM supplemented with 10% FBS and 1% penicillin–streptomycin (basal medium II, BMII), and the medium was changed every two days thereafter [38]. During differentiation, cells were treated with different concentrations of *FYGL* (0, 50, 100, 200, 400, and 800 μg/mL). Undifferentiated cells cultured in BMI were used as the blank control group, and differentiated cells cultured in BMI without *FYGL* were used as the model groups.

### Uptake of *FYGL* in 3T3-L1 cells

Three milligrams of FITC fluorescence agent was dissolved in 0.3 mL of DMSO to prepare a FITC solution with a concentration of 10 mg/mL, and then the solution was diluted to 1 mg/mL with sodium buffer (SB). *FYGL* (10 mg) was dissolved in 10 mL SB to form a 1 mg/mL *FYGL* solution, which was mixed with the diluted FITC solution at a volume ratio of 10: 1. The mixture was stirred at low temperature (ice bath) to allow the formation of fluorescent FITC–*FYGL* complexes. After the coupling reaction proceeded overnight, the solution was dialyzed with a 1 kDa dialysis bag to filter free FITC and then cryodesiccated.

3T3-L1 cells were seeded on microscope cover glasses in a 24-well plate with a density of 1 × 10^4^ cells per well and were incubated with FITC–*FYGL* complex (200 μg/mL) for 4 h. Nuclei and F-actin (filamentous actin) in 3T3-L1 cells were stained by DAPI and phalloidin-TRITC (phalloidin-tetramethyl rhodamine), respectively. Cell images were acquired with a C2^+^ laser scanning confocal microscope (Nikon, Japan). Moreover, 3T3-L1 cells were treated with the indicated concentrations of FITC–*FYGL* (0, 50, 100, 200, 400, 800 μg/mL) for 4 h, and then the fluorescence intensity was determined by flow cytometry (Gallios, Beckman Coulter) to visualize the uptake of *FYGL* in the cells.

### Measurement of cell viability

Cell viability was measured by a cell counting kit-8 (CCK-8) assay. In brief, 3T3-L1 cells were plated into 96-well plates with a density of 5 × 10^3^ cells per well and incubated to near confluence. Some cells were incubated in DMI with different concentrations of *FYGL* (0, 100, 200, 400, and 800 μg/mL) for 24 h. After treatment for 24 h, the medium was discarded, and fresh DMI containing CCK-8 solution was added to the 96-well plates. Approximately 1 h later, a multimode microplate reader (Cytation3, BioTek, U.S.A.) was used to measure the optical density (OD) at 450 nm.

### Triglyceride quantification

Triglyceride (TG) concentrations were determined using a commercial kit (Jiancheng Bioengineering Institute, China). Briefly, differentiated 3T3-L1 cells were washed twice with phosphate-buffered saline (PBS) and harvested by scraping from the culture plate in PBS containing 1% Triton X-100 on Day 6. Cell homogenates were obtained by sonication, and TG concentrations were determined using a commercial kit according to the manufacturer's instructions. Protein concentrations were measured using the bicinchoninic acid (BCA) protein assay kit (Beyotime, China) and used for quantification of proteins in samples.

### Oil red O staining and quantification

Lipid accumulation in cells was measured by oil red O staining. Differentiated 3T3-L1 cells were subjected to oil red O staining with modified oil red O staining kits (Beyotime, China). Briefly, the cells were washed with phosphate-buffered saline (PBS, pH 7.4) and then fixed with 10% (v/v) paraformaldehyde at room temperature for 10 min. Then, the fixation solution was removed, and the cells were washed twice with PBS. The cells were immersed in washing solution for 20 secs. After the washing solution was discarded, modified oil red O was added and incubated with the cells at room temperature for 20 min. Then, the staining solution was removed, and the cells were washed with washing solution once and PBS twice. Finally, cells stained with oil red O were examined via a polarizing microscope (DM2500P, Leica, Germany). In addition to this gross evaluation, the dye was dissolved in 60% isopropanol solution, and the absorbance was measured at 510 nm for quantification of lipid accumulation.

### RNA extraction and RT‒qPCR analysis

Total RNA was isolated from differentiated 3T3-L1 cells using RNAprep pure cell kits (TIANGEN, China) according to the manufacturer's instructions. Conversion of total RNA to single-stranded cDNA was performed using HiScript III All-in-one RT SuperMix Kits (Vazyme, China). The series of primers of β-actin (as an internal reference), C/EBPα, FABP4, ATGL, and LPL shown in Table 1 were synthesized by Sangon Co. The primers were mixed with cDNA templates, and qPCR was then performed with a Taq Pro Universal SYBR qPCR Master Mix kit (Vazyme, China) on a qPCR instrument (Bio-Rad, Germany) to amplify the DNA of C/EBPα, FABP4, ATGL, and LPL. The melt curves of the cDNA were analysed to determine the specificity of amplification, and quantification of relative mRNA levels was performed using the 2^−ΔΔCt^ method with normalization to β-actin mRNA.

**Table 1:** The primer sequences

Gene	Forward primer (5’ to 3’)	Reverse primer (5’ to 3’)
β-actin	GGGAATGGGTCAGAAGGACTC	GGTGTGGTGCCAGATCTTCTC
C/EBPα	GAAGGTGCTGGAGTTGACCAG	CCTTGACCAAGGAGCTCTCAG
FABP-4	AAGGTGAAGAGCATCATAACCCT	TCACGCCTTTCATAACACATTCC
ATGL	ATGTTCCCGAGGGAGACCAA	GAGGCTCCGTAGATGTGAGTG
LPL	TTGCCCTAAGGACCCCTGAA	TTGAAGTGGCAGTTAGACACAG

### Protein extraction and immunoblot analysis

Immunoblot analysis was performed according to the method described in a previous report with a minor modification [39]. Differentiated 3T3-L1 cells were lysed in RIPA lysis buffer and centrifuged (12,000 × *g*, 10 min, 4℃). Proteins in the lysates were separated by 10% SDS‒PAGE and transferred to polyvinylidene fluoride membranes. Then, the membranes were blocked in TBST/5% nonfat dry milk powder; incubated overnight at 4 °C with antibodies against FABP4, PPARγ, CEBPα, AMPKα, p-AMPKα, ATGL, HSL, LPL, and β-actin; and incubated with a goat anti-rabbit secondary antibody at room temperature for 1 h. Finally, enhanced chemiluminescence solution (ECL) was used to detect the proteins on the membranes. The luminescence signals were recorded with a Chemiscope3300 mini (Clinx Science Instruments, China). Data were collected from three independent experiments.

### Statistical analysis

All data were analysed by SPSS 20.0 (SPSS, Inc., U.S.), and expressed as the mean ± S.D. values. All the data were significantly drawn from a normally distributed population at the 0.05 level, justified by Shapiro–Wilk normality test. One-way ANOVA followed by the Bonferroni correction was performed to analyse the statistical significance of differences among the groups. A value of *P* < 0.05 was considered statistically significant.

## Results

### Effect of *FYGL* on histopathology of beige adipose tissue in vivo

**Fig. 3:**
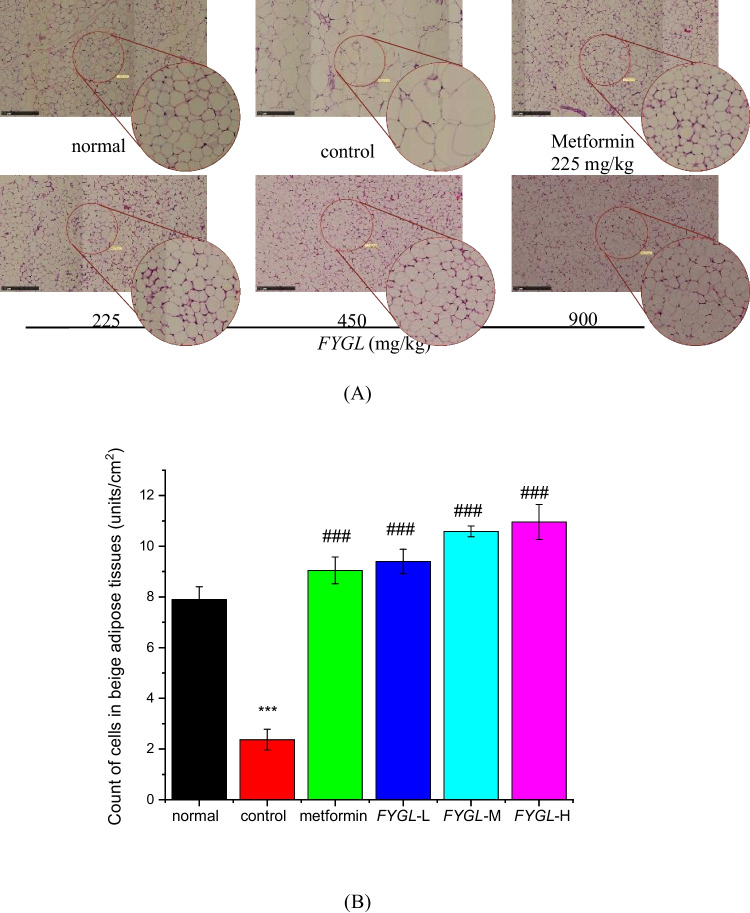
Histopathological analysis of adipocytes in beige adipose tissues. **A** Representative images of H&E-stained beige adipose tissues, magnification 100 × . The scale bar represents 250 μm. **B** Semiquantitative analysis of the adipocyte number per area in beige adipose tissue by Image-Pro Plus 6.0 software. The mean ± S.D. values are presented (*n* = 6; ^***^P < 0.001 *vs.* normal; ^#^*P* < 0.05, ^##^*P* < 0.01, ^###^*P* < 0.001 *vs.* control)

In the present work, beige adipose tissue in *db/db* mice was subjected to histopathological analysis. Figure 3A shows that the size of beige adipocytes was larger, and the numbers were lower in the control group than in the normal group, whereas treatment with metformin and *FYGL* reduced the size of adipocytes. Semiquantitative analysis of H&E staining in Fig. 3B showed that *FYGL* significantly increased the number of adipocytes per unit area of beige adipose tissue in a dose-dependent manner.

### Effect of *FYGL* on lipid metabolism in vivo

**Fig. 4:**
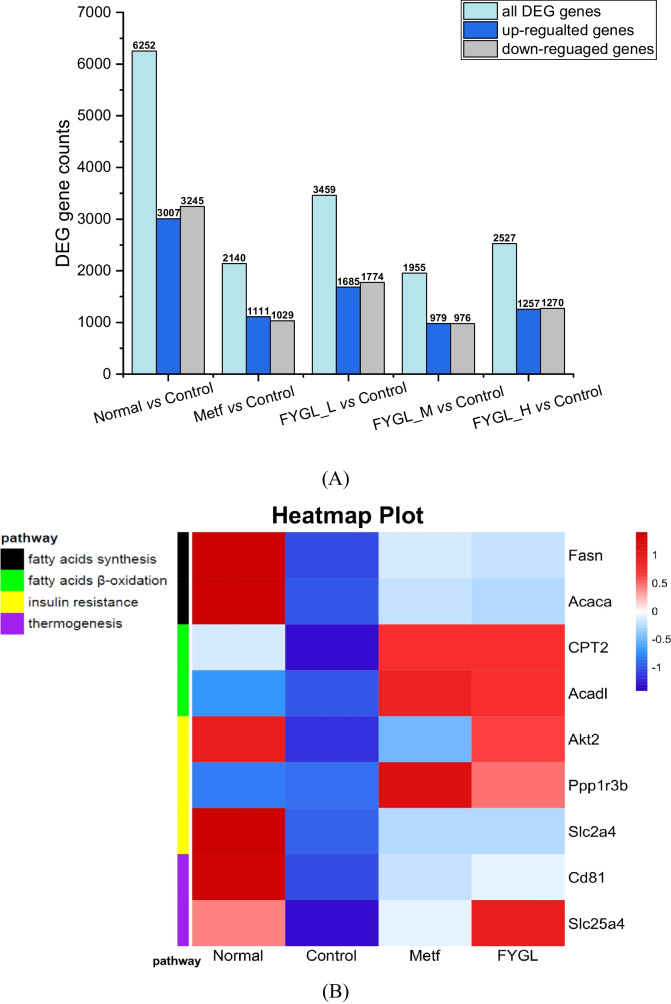
Transcriptome analysis of RNA sequencing of beige adipose tissue in the normal, metformin, and *FYGL* groups compared to the control group. **A** DEG counts. **B** Hierarchical clustering heatmap of the expression profile of the DEGs

Type 2 diabetes is strongly associated with genes of lipid metabolism [40]. In this work, beige adipose tissue transcriptome sequencing was performed to explore the potential molecular mechanism of lipid metabolism in vivo. As shown in Fig. 4A, the screening results of the differentially expressed genes (DEGs) showed that the ratio of upregulated: downregulated: all differentially expressed genes was approximately 0.5: 0.5: 1 in the metformin and *FYGL* groups compared with the control group, nearly the same as the ratio in the normal group compared to the control group. Figure 4B shows the hierarchical clustering heatmap. The large coloured square patterns represent the upregulated or downregulated genes in the different groups. The change in colour from blue to red indicates a change in the gene expression from downregulation to upregulation. The narrow columns on the left show the pathway-related genes. Figure 4B shows that the colour patterns of the DEGs in the control group were different from those in the normal group for most genes except *Ppp1r3b*, while the colour patterns in the *FYGL* group were similar to those in the normal group. From the pathway indication in the upper-left corner in Fig. 4B, it can be seen that the DEGs were involved in the pathways of fatty acid synthesis (black), fatty acid oxidation (green), insulin resistance (yellow), and thermogenesis (purple).

As shown in Fig. 4B, *FYGL* increased the mRNA levels of *Ppp1r3b*, *Fasn*, *Acaca*, *CPT2*, and *Acadl* in the beige adipose tissue of *db/db* mice compared to those in the control group. The *Ppp1r3b* gene encodes protein phosphatase 1, which is a critical protein in glycogen metabolism regulated by insulin [41]. The *Fasn* (encoding FAS [42]) and *Acaca* (encoding ACCα [43]) genes are involved in fatty acid synthesis [42, 43]. *CPT2* (encoding CPT-II, carnitine palmitoyl transferase II [44]) and *Acadl* (encoding ACADL, acyl-CoA dehydrogenase long chain [45]) are involved in the β-oxidation of long-chain fatty acids in mitochondria [44, 45]. The imbalance between fatty acid synthesis and degradation can lead to dyslipidaemia, diabetes and cardiovascular disease [46, 47]. Transcript analysis of those genes in beige adipose indicated that *FYGL* could upregulate fatty acid metabolism in vivo. Additionally, as shown in Fig. 4B, *FYGL* upregulated fatty acid degradation genes (*CPT2* and *Acadl*) more significantly than fatty acid synthesis genes (*Fasn* and *Acaca*). In addition, as shown in Fig. 4B, *FYGL* increased the levels of *Cd81* (encoding CD81 [48]) and *Slc25a4* (encoding SLC25A4 [49]) compared to those in the control group, and the levels of these mRNAs in the *FYGL* group were even higher than those in the metformin group. CD81 is a marker of beige adipocyte progenitors. The absence of CD81 leads to diet-induced obesity, insulin resistance, and adipose tissue inflammation [48]. SLC25A4, a mitochondrial ATP/ADP transporter, regulates thermogenesis of beige adipose through UCP1-independent mechanisms [50]. Beige adipocytes can produce heat by metabolizing fatty acids. Transcriptome analysis indicated that *FYGL* could increase the expression of thermogenesis genes (*Cd81* and *Slc25a4*) in beige adipose, as indicated by the transition from blue to red in Fig. 4B. Furthermore, *FYGL* and metformin increased the mRNA levels of *Akt2* (encoding AKT2 [51]) and *Slc2a4* (encoding GLUT-4, glucose transporter-4 [52]), as shown in Fig. 3B. Deficiency of AKT2 and GLUT-4 leads to type 2 diabetes and insulin resistance [51, 53]. The relative study and results have been reported by Pan et al. (see Figure S1 and S2 in Supplementary materials) [34, 54].

**Fig. 5:**
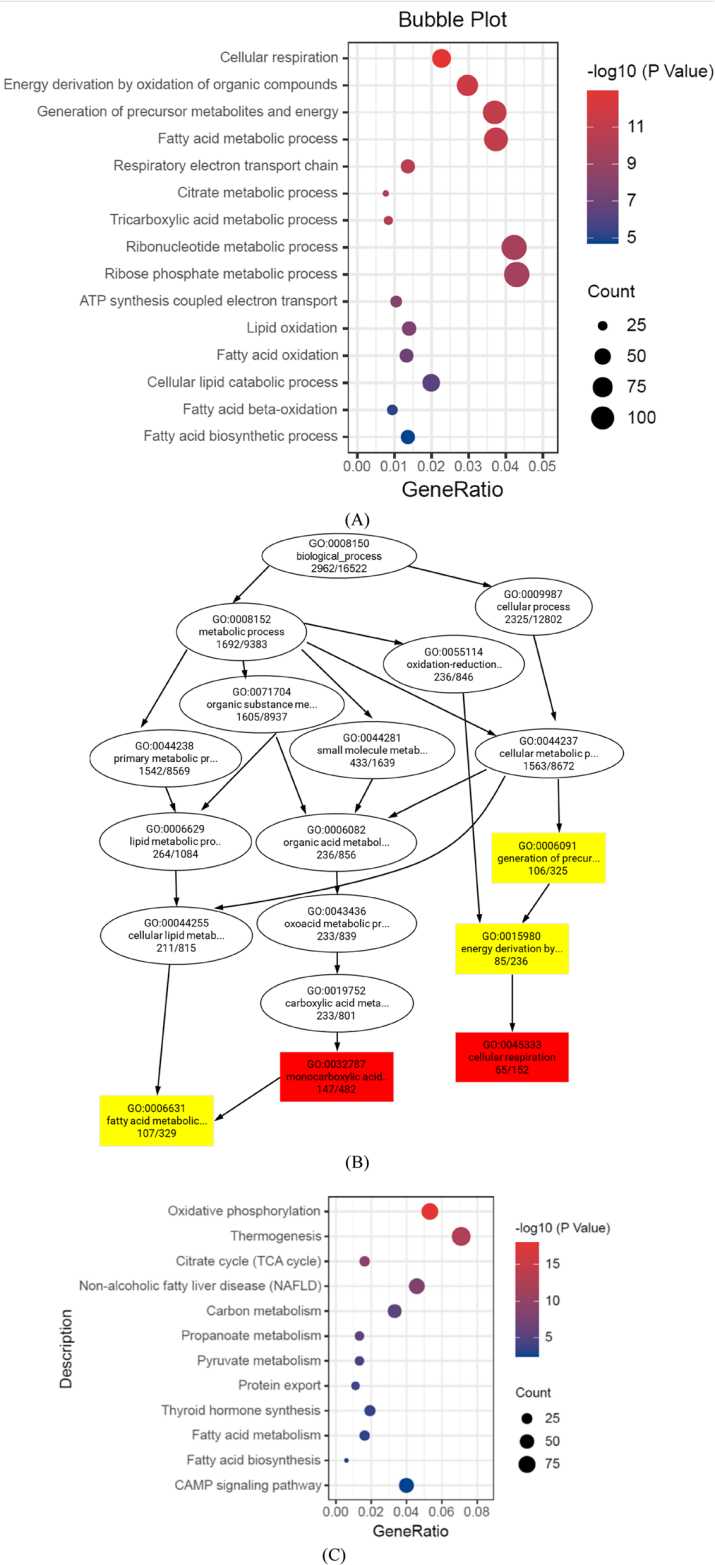
GO and KEGG functional enrichment analyses based on the DEGs. **A** Bubble plot of biological processes in the GO enrichment analysis. **B** The directed acyclic graph of biological process in the GO enrichment analysis. **C** Bubble plot of pathways in the KEGG enrichment analysis

The GO (Gene Ontology) database is a comprehensive database describing gene functions, which includes the biological process (BP), cellular component (CC), and molecular function (MF) in ontological categories. Figure 5A shows the bubble plot of the biological processes in the Gene Ontology (GO) enrichment analysis (*FYGL vs.* control), where the redder the dot is, the more significant the enrichment of the biological process. Figure 5A shows that DEGs were mainly enriched in terms related to the biological processes of cellular respiration, fatty acid metabolism (gene *Fasn* and *Acaca*), tricarboxylic acid metabolism, fatty acid oxidation (gene *CPT2* and *Acadl*), etc., and that *FYGL* restored beige adipose functions in *db/db* mice through those biological processes. Figure 5B is a directed acyclic graph (DAG, *FYGL vs.* control) of the GO biological process enrichment analysis, which indicates the relationship of functions from up- to down- biological processes. Figure 5B shows that the biological processes were eventually refined to fatty acid metabolism and cellular respiration.

Figure 5C shows the bubble plot of the signalling pathways in the Kyoto Encyclopedia of Genes and Genomes (KEGG) enrichment analysis (*FYGL vs.* control), where the redder the dot is, the more significant the enrichment of the signalling pathway. Figure 5C shows that DEGs were predominantly enriched in signalling pathways related to oxidative phosphorylation, thermogenesis, citrate cycle (TCA cycle), fatty acid metabolism, and fatty acid biosynthesis. The data in Fig. 5C suggest that *FYGL* promotes the functions of beige adipose through those signalling pathways.

### Cellular uptake of *FYGL*

**Fig. 6:**
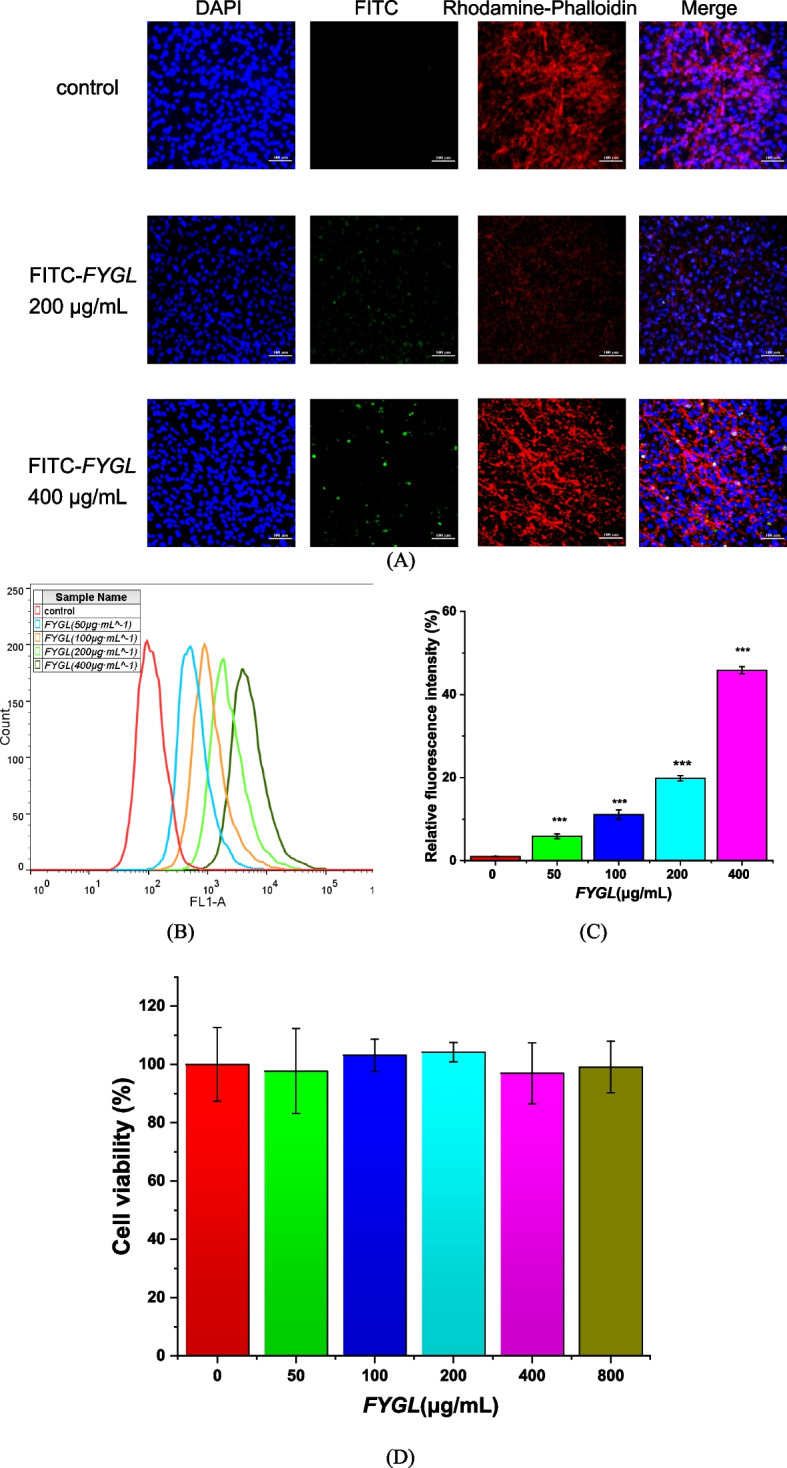
**A** Laser confocal scanning microscopy images of *FYGL* in 3T3-L1 cells at 200 × magnification. 3T3-L1 cells were incubated with FITC–*FYGL* (200 μg/mL) for 4 h; blue (DAPI labelled), red (rhodamine labelled) and green (FITC labelled) represent the nucleus, cytoskeleton and *FYGL*, respectively. The scale bar represents 100 μm. **B** Flow cytometric analysis of fluorescence. **C** Geometric means calculated by FlowJo software. The data are presented as the mean ± S.D. values (*n* = 3). ^***^P < 0.001 *vs.* control group. **D** Effect of *FYGL* on cell viability. 3T3-L1 cells were incubated with various concentrations of *FYGL* (0, 50, 100, 200, 400 and 800 μg·mL.^−1^) for 24 h, and cell viability was determined by a CCK-8 assay. The mean ± S.D. values are presented (*n* = 6)

To reveal the underlying mechanisms of *FYGL* in mediating biological functions, investigations at the cellular level are necessary. Figure 6A shows the uptake of *FYGL* (200 μg/mL and 400 μg/mL) in 3T3-L1 cells, as measured by confocal laser scanning microscopy, where green fluorescence was found in the cells cultured with FITC labelled-*FYGL*, indicating that *FYGL* could be taken up well into 3T3-L1 cells. Moreover, the results of flow cytometric analysis of *FYGL* uptake in 3T3-L1 cells are shown in Fig. 6B and C; the peak of the curve shifted to the right as the FITC-*FYGL* concentration increased, and the uptake of *FYGL* in cells occurred in a dose-dependent manner.

### Effect of *FYGL* on cell viability

To examine the cytotoxicity of *FYGL* in 3T3-L1 adipocytes, cell viability was measured using the CCK-8 assay. Adipocytes were treated with various concentrations of *FYGL* (0 − 800 μg/mL). The CCK-8 assay results shown in Fig. 6D demonstrate that *FYGL* had no obvious cytotoxicity at concentrations up to 800 μg/mL.

### Effect of *FYGL* on the accumulation of intracellular triglycerides and lipids

**Fig. 7:**
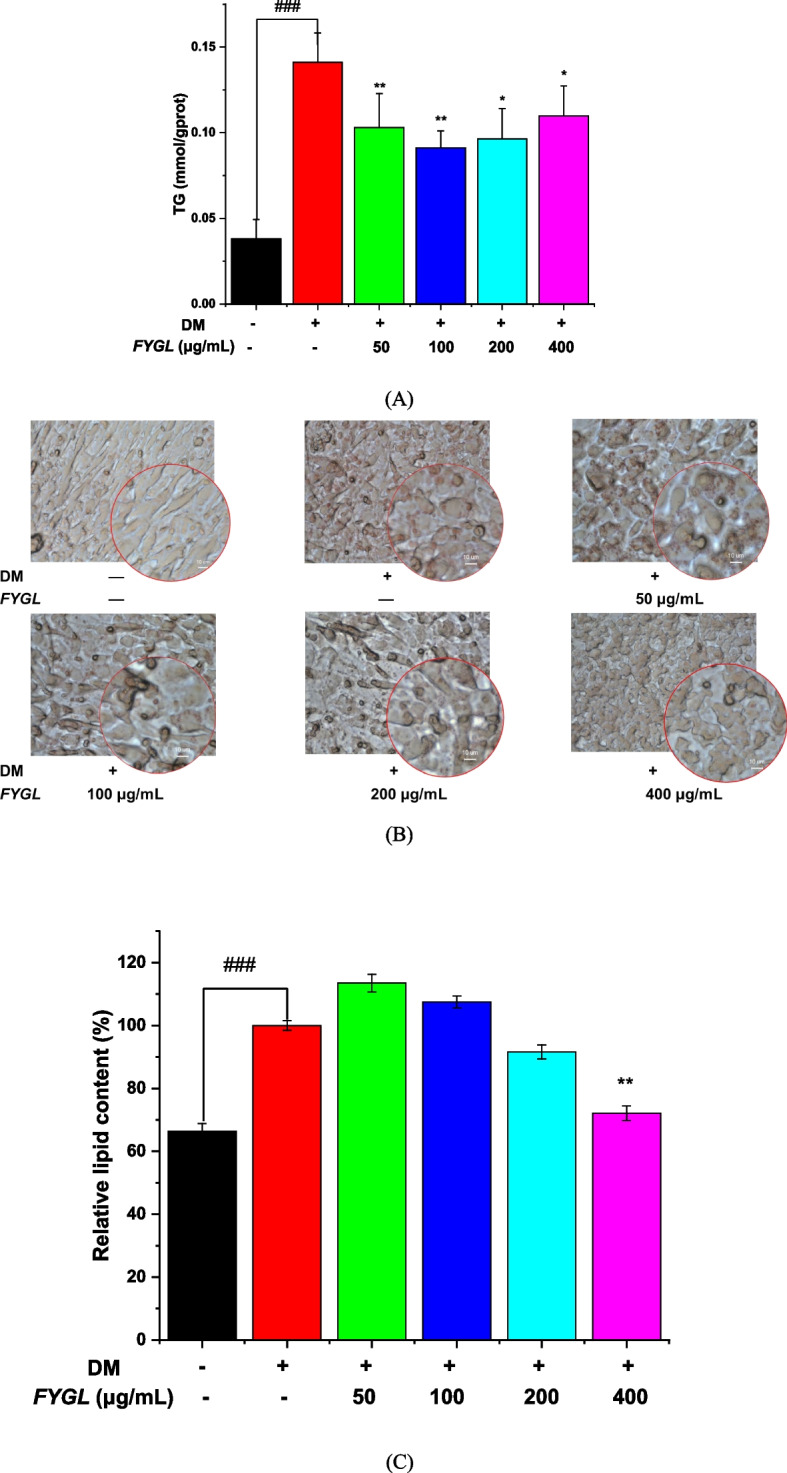
Effect of *FYGL* on the inhibition of lipid accumulation in mature adipocytes. Differentiated 3T3-L1 cells were incubated with *FYGL* at concentrations ranging from 0–400 μg/mL. **A** Intracellular TG in mature adipocytes. **B** Intracellular lipid droplets stained by oil red O and visualized by polarized phase contrast microscopy (500 ×). **C** Intracellular lipid accumulation was quantitatively measured using a microplate reader at an absorbance of 490 nm. Mean ± S.D. values are presented (*n* = 6). ^###^*P* < 0.001 *vs.* blank control group, ^**^P < 0.01, ^*^P < 0.05 *vs.* model group

Lipid accumulation in adipocytes is a hallmark of adipogenesis. Excessive accumulation of lipid droplets in adipocytes leads to obesity and insulin resistance [16]. Mature differentiated cells accumulate triglycerides, which then converge to form lipid droplets (LDs). Figure 7A shows that *FYGL* significantly decreased the triglyceride content. Moreover, cell differentiation and lipid accumulation can be identified by oil red O staining and triglyceride assays. Figure 7B shows that the number of lipid droplets (red staining) was markedly increased in cells cultured in differentiation medium (DM) but was significantly decreased when the cells were cultured with *FYGL* (200 − 400 μg/mL), and Fig. 7C quantitatively shows the effect of *FYGL* on lipid droplet accumulation. *FYGL* inhibited triglyceride accumulation and lipid droplet aggregation in differentiated adipocytes. The mechanism of inhibition was further investigated as follows.

### Effect of *FYGL* on the expression of adipogenic and lipolytic genes and proteins

**Fig. 8:**
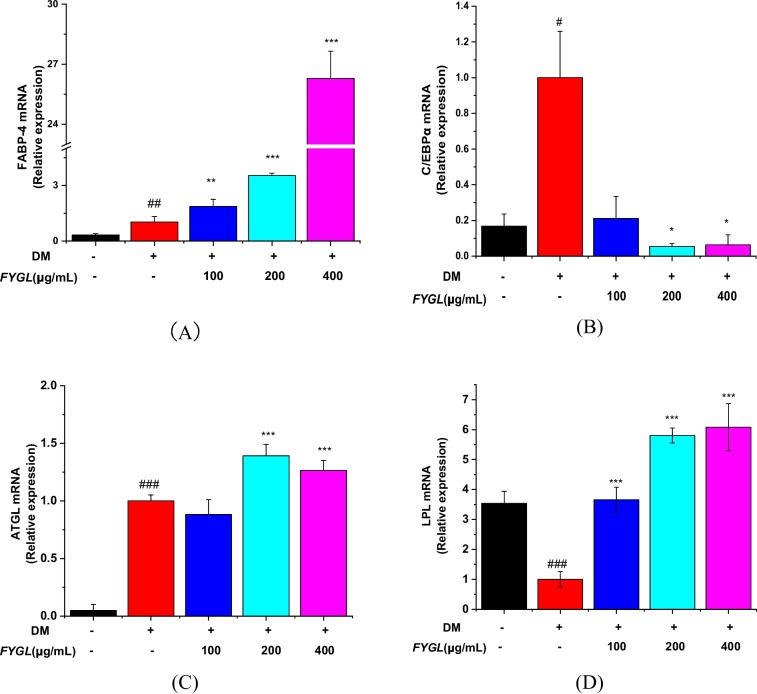
The relative mRNA expression levels of (**A**) C/EBPα, (**B**) FABP-4, (**C**) ATGL, and (**D**) LPL in differentiated 3T3-L1 cells, with reference to the model group. Data are presented as the mean ± S.D. values (*n* = 6). ^###^*P* < 0.001, ^##^*P* < 0.01, ^#^*P* < 0.05 *vs.* blank control group. ^***^P < 0.001, ^**^P < 0.01, ^*^P < 0.05 *vs.* model group

Several reports have shown that peroxisome proliferator-activated receptor γ (PPARγ) and CCAAT/enhancer-binding protein α (C/EBPα) are marker proteins of adipocyte differentiation and adipogenesis [12, 55, 56]. To reveal the mechanisms by which *FYGL* inhibits the accumulation of intracellular triglycerides and lipids, the effect of *FYGL* on the expression of adipogenic and lipolytic genes and proteins was investigated. Figure 8A shows that *FYGL* significantly increased the transcript level of FABP-4 in 3T3-L1 preadipocytes and considerably increased the transcript level of C/EBPα in adipocytes cultured in differentiation medium (Fig. 8B). Moreover, *FYGL* increased the mRNA level of lipolytic genes, such as ATGL (Fig. 8C) and LPL (Fig. 8D).

**Fig. 9:**
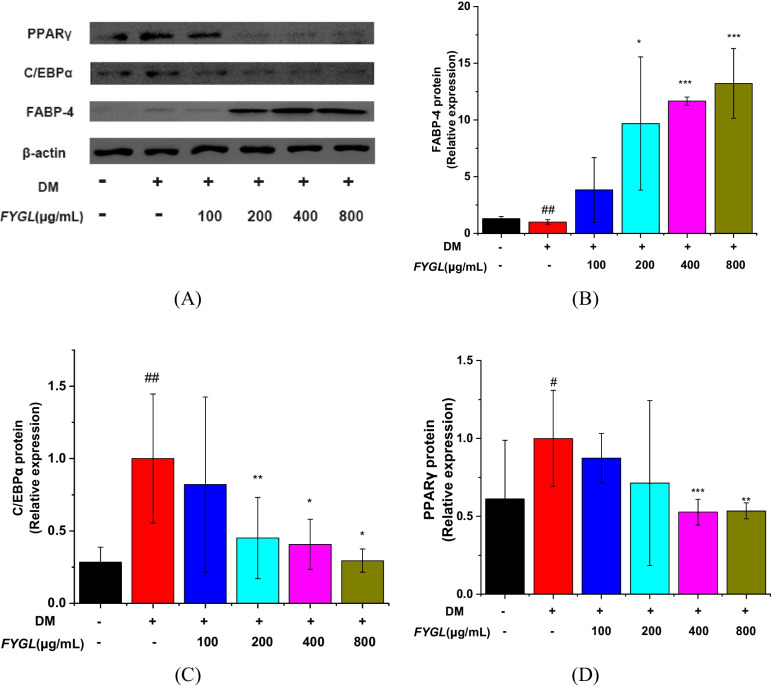
Western blot analysis of proteins involved in cellular differentiation in mature 3T3-L1 cells. **A** Images of the PPARγ, C/EBPα, and FABP-4 protein bands relative to the β-actin protein band. **B**, **C** and **D** Relative expression of PPARγ, C/EBPα, and FABP-4, respectively, with reference to β-actin, and normalized to the model group. Data are presented as the mean ± S.D. values (*n* = 3). ^##^*P* < 0.01, ^#^*P* < 0.05 *vs.* blank control group, ^***^P < 0.001, ^**^P < 0.01, ^*^P < 0.05 *vs.* model group

Furthermore, Western blotting was used to analyse the protein expression of FABP-4, PPARγ, and C/EBPα, as shown in Fig. 9A. *FYGL* greatly increased FABP-4 expression, as shown in Fig. 9B, and markedly decreased PPARγ and C/EBPα expression, as shown in Fig. 9C and D.

### Effect of *FYGL* on lipid metabolism and the AMPKα signalling pathway

**Fig. 10:**
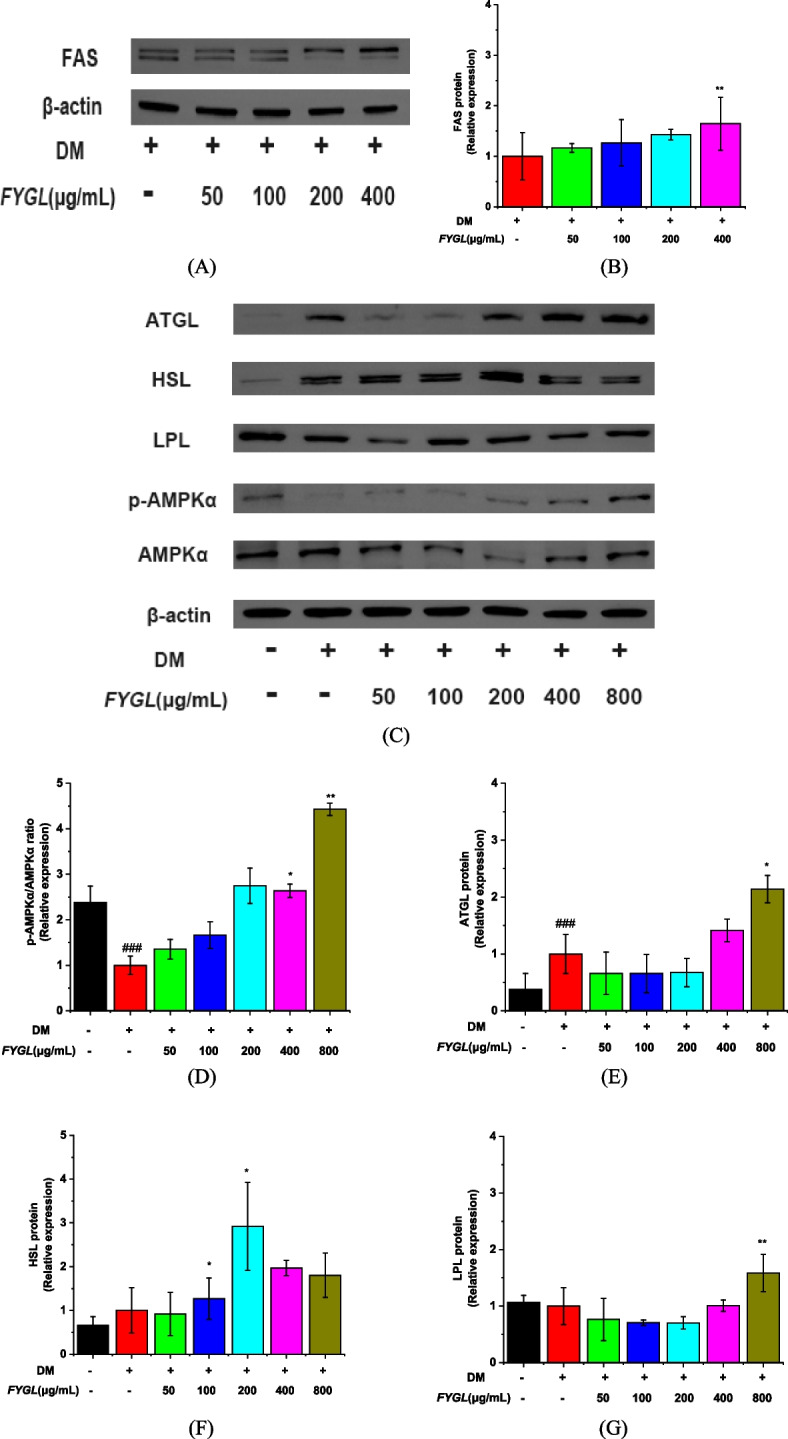
Western blot analysis of proteins involved in lipolysis and the AMPKα signalling pathway in mature 3T3-L1 cells. **A** Image of FAS protein bands, **B** Quantification of FAS expression. **C** Images of ATGL, HSL, LPL, p-AMPKα, and AMPKα protein bands. **D**, **E**, **F** and **G** Quantification of ATGL, HSL, LPL, and p-AMPKα/AMPKα protein levels, respectively. The protein levels in the model group are normalized to a value of 1.0. Data are presented as the mean ± S.D. values (*n* = 3). ^##^*P* < 0.01, ^###^*P* < 0.001 *vs.* blank control group, ^**^P < 0.01, ^*^P < 0.05 *vs.* model group

The fatty acid synthase (FAS) and AMPKα signalling pathway play important roles in lipogenic pathways [57]. Activating the AMPK signalling pathway can increase the activity of the lipases of ATGL and HSL, thus promoting the utilization of lipid storage [19, 58–60]. As shown in Fig. 10A and B, *FYGL* increased the protein expression of FAS. Additionally, *FYGL* increased the phosphorylation of AMPKα (Fig. 10C & D) and consequently increased the protein expression of lipolysis markers, such as ATGL (Fig. 10E), HSL (Fig. 10F), and LPL (Fig. 10G).

In addition, *FYGL* increased the protein levels of ATGL (Fig. 10E) and HSL (Fig. 10F) by twofold compared with that of FAS (Fig. 10B) and by 1.5-fold compared with those in the control group at concentrations higher than 200 μg/mL. Therefore, *FYGL* upregulated lipolysis more significantly than fatty acid biosynthesis, consistent with the results in vivo.

## Discussion

Cypess et al. proved that the amount and activity of beige adipose tissue are inversely correlated with body mass index [61]; the smaller the size and the greater the number of beige adipocytes, the healthier the body [62–64]. Ouellet et al. demonstrated that the activity of beige adipocytes is positively correlated with the level of glucose uptake in cells, which modulates the blood glucose content [65]. Therefore, increasing the number or activation of beige adipocytes could be a potential approach to treat type 2 obesity-induced diabetes [66]. Consistent with those studies, the results of this study showed that beige adipocytes were significantly enlarged in size and decreased in number in *db/db* mice, while these changes were significantly reversed after *FYGL* treatment.

Based on GO database of gene functions and KEGG enrichment analysis of signalling pathways, the results indicated that *FYGL* upregulated the fatty acid metabolism process and promoted thermogenesis in beige adipocytes. In addition, *FYGL* could balance fatty acid biosynthesis and metabolism to effectively dissipate energy, therefore reducing insulin resistance and increasing insulin sensitivity in vivo.

Tali et al. found that fatty acid binding protein-4 (FABP-4)-null preadipocytes can enhance PPARγ expression and activity, while the overexpression of FABP-4 inhibits PPARγ expression and adipogenesis [67]. Furuhashi et al. further found that FABP-4-null mice exhibit decreased lipolysis in adipocytes and pancreatic β cells and reduced insulin secretion [68]. The present work proved that *FYGL* could inhibit the differentiation of 3T3-L1 preadipocytes and promote lipolysis in mature adipocytes, therefore reducing lipid droplet accumulation. Studies have shown that fatty acid synthase (FAS) plays an important role in lipogenic pathways, which are involved in fatty acid biosynthesis [57]. In addition, the AMPKα signalling pathway also plays a critical role in lipolysis [19, 58, 60]. ATGL and HSL catalyse triglyceride degradation, releasing the fatty acids in lipid droplets of adipocytes [69], while adipocytes secrete LPL to degrade triglycerides in very low-density lipoprotein (VLDL) in vessels [70]. The results of present study indicated that *FYGL* promoted the degradation of lipid droplets in mature adipocytes by activating the AMPKα signalling pathway. Taken together, the results of the study on the cellular level showed that *FYGL* could inhibit lipid accumulation by both suppressing the differentiation of preadipocytes and promoting the degradation of lipid droplets in mature adipocytes to alleviate metabolically unhealthy obesity.

### Strengths and limitations of *FYGL* compared with reported work

In recent years, some natural medicinal plants have been used in the treatment of obesity and metabolic diseases because of their safety [71]. *Hibiscus rosa-sinensis* flowers were reported to be capable of decreasing obesity by reducing adipogenesis and activating AMPK to promote fatty acid oxidation [11]. *Momordica charantia* extracts can activate the AMPK signalling pathway, reduce adipogenic gene expression and peroxisome proliferator-activated receptor (PPAR) signalling in adipose tissue, and increase lipid oxidation in adipose tissue, thereby reducing obesity and insulin resistance [72, 73]. In addition, cannabidiol can promote adipocyte browning for the treatment of metabolic diseases [74].

Because *FYGL* is a macromolecular proteoglycan, it has multiple diabetes related-targets, such as protein tyrosine phosphatase 1B (PTP1B, a receptor of insulin resistance [31]), α-glucosidase [36] and amylin [75] through π-π stacking between aromatic groups in proteins as well as hydrogen bonding between protein and saccharide. These properties provide the basis of *FYGL* efficacy. *FYGL* also has anti-oxidation because of its reductive sugar residues. Therefore, *FYGL* play multiple roles by multiple targets on alleviating blood glucose [28], improving the lipid metabolism [54], reducing the diabetes-induced reactive oxygen species [37] and regulating immunity [76], in addition to specific action against obesity-induced diabetes. Importantly, *FYGL* is highly safe with LD50 of 6 g/kg [31], much safer than clinic anti-obesity drugs, such as orlistat and liraglutide. *FYGL* is an ingredient of macromolecules with a molecular weight distribution, which limits one to characterize its molecular structure as clearly as small molecules but could allow it to act multiple functions.

It should be mentioned that *FYGL* is a macromolecular proteoglycan with multi-targets and multi-pathways to regulate obesity-induced diabetes. Its effective dose in clinic could not be converted simply from mice to human on the body weight like the common small molecular drug does. Especially for a targeting drug, different genus may have different responses and sensitivity. In addition, *FYGL* include amino acids and saccharides, consistent to those present in blood and urine, therefore, it is difficult to follow the tracks of *FYGL* when it was orally administrated and possibly decomposed in stomach. Previously, we studied the distribution of Cy5-labled *FYGL* in mice and found that *FYGL* was available in small intestine and viscera, and enriched mostly in liver, and excreted from the body after 12 h [76].

## Conclusion

**Fig. 11:**
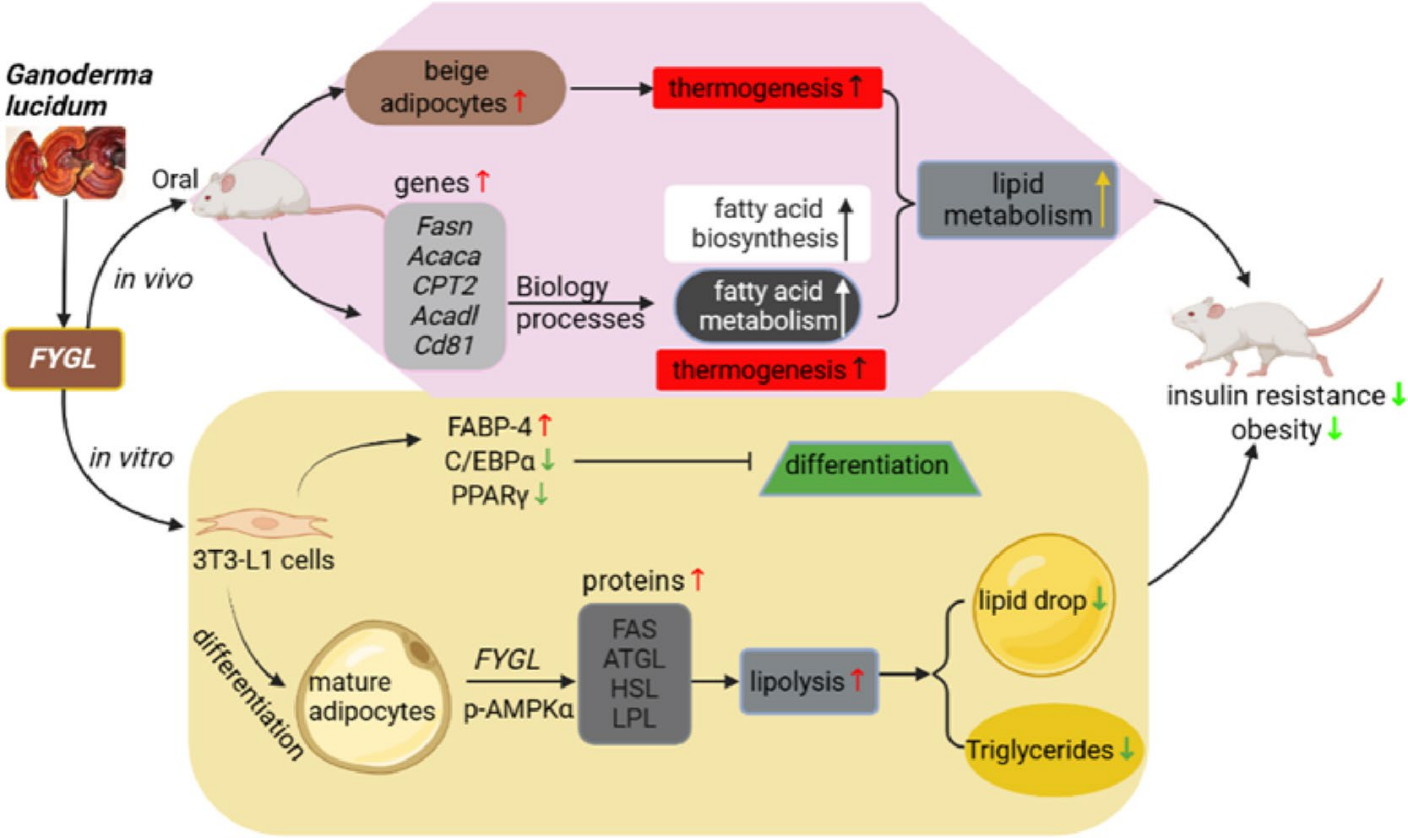
Profile of the mechanism of *FYGL* in ameliorating obesity-induced diabetes

This study showed that *FYGL* could increase the number of beige adipocytes and restore adipocyte morphology, thereby alleviating metabolic disorders in *db/db* mice. The mechanism in vivo by which *FYGL* alleviates metabolic disorders involves the balance between fatty acid biosynthesis and metabolism to effectively dissipate energy in beige adipocytes. In vitro, *FYGL* inhibited the differentiation of preadipocytes by increasing FABP-4 gene expression and decreasing PPARγ and C/EBPα gene levels. Moreover, *FYGL* promoted adipocyte browning by upregulating *Cd81* gene expression. Furthermore, *FYGL* increased the levels of the lipolysis-related proteins of ATGL, HSL and LPL by activating the AMPKα signalling pathway, therefore accelerating lipid metabolism in mature adipocytes. Importantly, these findings proved that *FYGL*, a proteoglycan, could modify metabolic disorders by targeting both preadipocytes and mature adipocytes. The mechanistic profile of *FYGL* in the treatment of obesity-induced diabetes is shown in Fig. 11. Hopefully, *FYGL* could be used as an agent to treat lipid metabolism disorders and obesity in the clinic.

### Supplementary Information

**Additional file 1.**

**Additional file 2.**

**Additional file 3.**

**Additional file 4.**

**Additional file 5.**

## Data Availability

The data presented in this study are available within the article.
