# Supplementary material for: Balancing adipocyte production and lipid metabolism to treat obesity-induced diabetes with a novel proteoglycan from Ganoderma lucidum
Source: Lipids Health Dis. 2023 Aug 8;22:120. doi: 10.1186/s12944-023-01880-6 (PMC10408226; doi:10.1186/s12944-023-01880-6)
Supplement: Supplementary file 1 — Additional file 1. [file 12944_2023_1880_MOESM1_ESM.docx]

**Supplementary materials**

**Balancing adipocyte production and lipid metabolism to treat obesity-induced diabetes with a novel proteoglycan from *Ganoderma lucidum***

YingXin Wang ^1^, Fanzhen Yu ^1^, Xinru Zheng ^1^, Jiaqi Li ^1^, Zeng Zhang ^2^, Qianqian Zhang ^1^, Jieying Chen ^1^, Yanming He ^2,*^, Hongjie Yang ^2,*^ and Ping Zhou ^1,*^.

^1^ State Key Laboratory of Molecular Engineering of Polymers, Department of Macromolecular Science, Fudan University, Shanghai 200433, China

^2^ Yueyang Hospital of Integrated Traditional Chinese and Western Medicine, Shanghai University of Traditional Chinese Medicine, Shanghai 200437, China

^*^ Correspondence to: pingzhou@fudan.edu.cn (P.Z.), Tel/Fax: +86-21-31244038; yanghongjie1964@aliyun.com (H.Y.); heyanming176@163.com (Y.H.)

|   A |   B |
| --- | --- |
|   C | |

Figure S1. Basic metabolic indexes of db/db mice treated with *FYGL* for 8 weeks. (A) blood glucose; (B) serum insulin level; (C) glycosylated hemoglobin. ^#^*p* < 0.05, ^##^*p* < 0.01, ***p* < 0.01 *vs.* control group [1].

|   A |   B |
| --- | --- |
|   C |   D |

Figure S2. Serum lipids profiles of db/db mice treated with *FYGL* for 8 weeks. (A) TG content; (B) TC content; (C) LDL-c content; (D) HDL-c content. **p* < 0.05, ***p* < 0.01, ****p* < 0.001 *vs.* control group [1].

**References**

1. Pan D, Zhang D, Wu J, Chen C, Xu Z, Yang H, Zhou P: **A novel proteoglycan from Ganoderma lucidum fruiting bodies protects kidney function and ameliorates diabetic nephropathy via its antioxidant activity in C57BL/6 db/db mice.** *Food and Chemical Toxicology* 2014, **63:**111-118.
