# Supplementary material for: Balancing adipocyte production and lipid metabolism to treat obesity-induced diabetes with a novel proteoglycan from Ganoderma lucidum
Source: Lipids Health Dis. 2023 Aug 8;22:120. doi: 10.1186/s12944-023-01880-6 (PMC10408226; doi:10.1186/s12944-023-01880-6)
Supplement: Supplementary file 2 — Additional file 2. [file 12944_2023_1880_MOESM2_ESM.pdf]

SPRINGER NATURE
Author Services

Editing Certificate

This document certifies that the manuscript

Balancing adipocyte production and lipid metabolism to treat diabetes-associated obesity with a novel proteoglycan from Ganoderma lucidum

prepared by the authors

YingXin Wang, Fanzhen Yu, Xinru Zheng, Jiaqi Li, Zeng Zhang, Qianqian Zhang, Jieying Chen, Yanming He, Hongjie Yang, Ping Zhou.

was edited for proper English language, grammar, punctuation, spelling, and overall style by one or more of the highly qualified native English speaking editors at SNAS.

This certificate was issued on **April 4, 2023** and may be verified on the SNAS website using the verification code **27AD-D5DD-7B5D-B182-AC66** .

Neither the research content nor the authors' intentions were altered in any way during the editing process. Documents receiving this certification should be English-ready for publication; however, the author has the ability to accept or reject our suggestions and changes. To verify the final SNAS edited version, please visit our verification page at secure.authorservices.springernature.com/certificate/verify.
If you have any questions or concerns about this edited document, please contact SNAS at support@as.springernature.com.

SNAS provides a range of editing, translation, and manuscript services for researchers and publishers around the world.
For more information about our company, services, and partner discounts, please visit authorservices.springernature.com.
