# Supplementary material for: Balancing adipocyte production and lipid metabolism to treat obesity-induced diabetes with a novel proteoglycan from Ganoderma lucidum
Source: Lipids Health Dis. 2023 Aug 8;22:120. doi: 10.1186/s12944-023-01880-6 (PMC10408226; doi:10.1186/s12944-023-01880-6)
Supplement: Supplementary file 3 — Additional file 3. [file 12944_2023_1880_MOESM3_ESM.pdf]

SPRINGER NATURE
Author Services

Springer Nature Author Services

Research Square AJE, LLC
601 W Main St, Ste 102
Durham, North Carolina, 27701, USA
Tax ID: 412141424

PAYMENT RECEIPT

Payment Date	**04 Apr 2023**
Reference Number	**2023040422001459441434093787**
Payment Mode	**Alipay**

Amount Received
CNY2,956.34

Received From

Ping Zhou
Fudan University, Jiangwan Campus, 2005 Songhu Road, Shanghai, China, 200438
Shanghai
200433
China
Ping Zhou
13817800753

Payment for

Invoice Number	Invoice Date	Invoice Amount	Payment Amount
SZH3B3RWG	04 Apr 2023	CNY2,956.34	CNY2,956.34
