# Supplementary material for: Balancing adipocyte production and lipid metabolism to treat obesity-induced diabetes with a novel proteoglycan from Ganoderma lucidum
Source: Lipids Health Dis. 2023 Aug 8;22:120. doi: 10.1186/s12944-023-01880-6 (PMC10408226; doi:10.1186/s12944-023-01880-6)
Supplement: Supplementary file 4 — Additional file 4. [file 12944_2023_1880_MOESM4_ESM.docx]

Suppl. Materials (Similarity index and explanation) for manuscript (ID d0b88a5c-0826-4e2b-aa1e-22db33dcbcf9, entitled as “Balancing adipocyte production and lipid metabolism to treat diabetes-associated obesity with a novel proteoglycan from Ganoderma lucidum”)

| Number | Similarity index (%) | Primary sources | Explanations |
| --- | --- | --- | --- |
| 1 | 5 | Ying Zhang, Yanna Pan, Jiaqi Li, Zeng Zhang, Yanming He, Hongjie Yang, and Ping Zhou. "Inhibition on α-Glucosidase Activity and Non-Enzymatic Glycation by an Anti-Oxidative Proteoglycan from Ganoderma lucidum", molecules, 2022. | The primary source is our published article. The checked overlaps are those in the sections of materials and methods, graphic descriptions, ethical statements, author contribution statements, and acknowledgements. |
| 2 | 4 | Haohui Liang, Yanna Pan, Yilong Teng, Shilin Yuan, Xiao Wu, Hongjie Yang & Ping Zhou. "A proteoglycan extract from Ganoderma Lucidum protects pancreatic beta-cells against STZ-induced apoptosis", Bioscience, Biotechnology, and Biochemistry, 2020. | as above. |
| 3 | 2 | Cihan Yang, Fei Wang, Doudou Huang, Haixia Ma, Lu Zhao, Guilin Zhang, Hailong Li, Qian Han, Dennis Bente, Zhiming Yuan, Han Xia. "Vector competence and transcriptional response of Aedes aegypti for Ebinur Lake virus, a newly mosquito-borne orthobunyavirus", 2022. | The checked overlaps with primary source are those in the sections of methods about RNA-seq. transcription, which are useful for our work, therefore cited. |
| 4 | 1 | Mengqing Wu, Dan Liu, Rong Zeng, Tao Xian, Yi Lu,  Guohua Zeng, Zhangzetian Sun, Bowei Huang, Qiren  Huang. "Epigallocatechin-3-gallate inhibits adipogenesis  through down-regulation of PPARγ and FAS expression  mediated by PI3K-AKT signaling in 3T3-L1 cells", European  Journal of Pharmacology, 2017 | as above. |
| 5 | 1 | Weipeng Qi, John M. Clark, Alicia R. Timme-Laragy, Yeonhwa Park. "Perfluorobutanesulfonic acid (PFBS) potentiates adipogenesis of 3T3-L1 adipocytes", Food and Chemical Toxicology, 2018. | as above. |
| 6 | 1 | Shilin Yuan, Yanna Pan, Zeng Zhang, Yanming He, Yilong Teng, Haohui Liang, Xiao Wu, Hongjie Yang, Ping Zhou. "Amelioration of the lipogenesis, oxidative stress  and apoptosis of hepatocytes by a novel proteoglycan from  <i>Ganoderma lucidum</i>", Biological and Pharmaceutical  Bulletin, 2020 | The primary source is our published article. The checked overlaps are those in the sections of materials and methods, graphic descriptions. |
| 7 | 1 | Bao-Song Teng, Chen-Dong Wang, Hong-Jie Yang, Jia-Sheng Wu, Dan Zhang, Min Zheng, Zhao-Hua Fan, Deng Pan, and Ping Zhou. "A Protein Tyrosine Phosphatase 1B Activity Inhibitor from the Fruiting Bodies of Ganoderma lucidum (Fr.) Karst and Its Hypoglycemic Potency on Streptozotocin-Induced Type 2 Diabetic Mice", Journal of Agricultural and Food, 2011 | as above. |
| 8 | 1 | Jing Zhou, Ji Zhang, Jiayi Li, Yiqiu Guan, Ting Shen, Fu Li, Xueqin Li, Xiaojun Yang, and Weicheng Hu. "Ginsenoside F2 Suppresses Adipogenesis in 3T3-L1 Cells and Obesity in Mice via the AMPK Pathway", Journal of Agricultural and Food, 2021 | Scientifically customary usage in reference. |
| 9 | 1 | Dong Xu, Tao Qiao, Yue Wang, Qiang-Song Wang, Yuan-Lu Cui. "Alginate nanogels-based thermosensitive hydrogel to improve antidepressant-like  effects of albiflorin via intranasal delivery", Drug Delivery, 2021 | As No. 3 |
| 10 | 1 | Jianghong Cheng, Jia Liang, Yingzhe Li, Xia Gao, Mengjun Ji, Mengying Liu, Yingpu Tian, Gensheng Feng, Wenbo Deng, Haibin Wang, Shuangbo Kong, Zhongxian Lu. "Shp2 in uterine stromal cells critically regulates on time embryo implantation and stromal decidualization by multiple pathways during early pregnancy", PLoS Genet, 2022 | As No.8 |
| 11 | 1 | Yanna Pan, Shilin Yuan, Yilong Teng, Zeng Zhang et  al. "Antioxidation of a proteoglycan from Ganoderma  lucidum protects pancreatic β-cells against oxidative stress induced apoptosis in vitro and in vivo", International Journal of  Biological Macromolecules, 2022 | As No. 1 |
| 12-16 | 1 |  | As No. 8 |
| 16-97 | <1 |  | As No. 8 |
