# Supplementary material for: Balancing adipocyte production and lipid metabolism to treat obesity-induced diabetes with a novel proteoglycan from Ganoderma lucidum
Source: Lipids Health Dis. 2023 Aug 8;22:120. doi: 10.1186/s12944-023-01880-6 (PMC10408226; doi:10.1186/s12944-023-01880-6)
Supplement: Supplementary file 5 — Additional file 5. [file 12944_2023_1880_MOESM5_ESM.pdf]

20230405201350841085309659541504

WORD COUNT	7764	TIME SUBMITTED	05-APR-2023 08:14PM
		PAPER ID	98394340

Balancing adipocyte production and lipid metabolism to treat diabetes-associated obesity with a novel proteoglycan from *Ganoderma lucidum*

YingXin Wang [1], Fanzhen Yu [1], Xinru Zheng [1], Jiaqi Li [1], Zeng Zhang [2], Qianqian Zhang [1], Jieying Chen [1], Yanming He [2,*], Hongjie Yang [2,*] and Ping Zhou [1,*].

[1] State Key Laboratory of Molecular Engineering of Polymers, Department of Macromolecular Science, Fudan University, Shanghai 200433, China

[2] Yueyang Hospital of Integrated Traditional Chinese and Western Medicine, Shanghai University of Traditional Chinese Medicine, Shanghai 200437, China

[*] Correspondence to: pingzhou@fudan.edu.cn (P.Z.), Tel/Fax: +86-21-31244038; yanghongjie1964@aliyun.com (H.Y.); heyanming176@163.com (Y.H.)

Abstract

Obesity is often accompanied by metabolic disorder and insulin resistance, resulting in type 2 diabetes. Based on previous findings, *FYGL*, a natural hyperbranched proteoglycan extracted from the *G. lucidum* fruiting body, can decrease blood glucose and reduce body weight in diabetic mice. In this article, the underlying mechanism of *FYGL* in ameliorating diabetes-associated obesity was further investigated both *in vivo* and *in vitro*. *FYGL* upregulated expression of metabolic genes related to fatty acid biosynthesis, fatty acid β-oxidation and thermogenesis; downregulated the expression of insulin resistance-related genes; and significantly increased the number of beige adipocytes in db/db mice. In addition, *FYGL* inhibited preadipocyte differentiation of 3T3-L1 cells by increasing the expression of FABP-4. *FYGL* not only promoted fatty acid synthesis but also more significantly promoted triglyceride degradation and metabolism by activating the AMPK signalling pathway, therefore preventing fat accumulation, balancing adipocyte production and lipid metabolism, and regulating metabolic disorders and unhealthy obesity. *FYGL* could be used as a promising pharmacological agent for the treatment of metabolic disorder-related obesity.

Keywords: metabolic disorder; obesity; diabetes; *Ganoderma lucidum*; adipocytes; lipid metabolism; 3T3-L1; AMPKα signalling pathway

Introduction

Type 2 diabetes mellitus (T2DM) is a chronic degenerative disease, and 60% of T2DM patients are obese as a result of metabolic disorder and insulin resistance as well as impaired energy homoeostasis [1-6]. Adipose tissues play an important role in surplus energy storage and energy metabolism [7]. Adipose tissue comprises white adipose tissue (WAT) and brown or beige adipose tissue (BAT). WAT mainly functions to store fat in the form of lipid droplets and secrete adipokines to regulate the metabolism of tissues such as muscle and liver tissues [8]. BAT mainly functions to dissipate excess energy through thermogenesis to maintain a stable body weight, and it secretes many batokines to affect the physiology of a variety of organ systems and tissues, such as the liver, heart and muscle [9,10]. Accumulating evidence has suggested that a high ratio of white to beige adipocytes is associated with insulin resistance [3,5].

Adipocytes are differentiated from preadipocytes; therefore, many studies have focused on inhibiting the differentiation of preadipocytes in addition to lipid metabolism to treat obesity [11,12]. Mesenchymal stem cells (MSCs) undergo a two-step process to differentiate into adipocytes: MSCs first differentiate into preadipocytes, and preadipocytes continue to differentiate into mature adipocytes [13,14]. During adipogenesis, peroxisome proliferator-activated receptor gamma (PPARγ) and CCAAT/enhancer-binding protein α (C/EBPα) are marker proteins for preadipocytes differentiating into mature adipocytes [15,16]. Subsequently, fatty acids are synthesized in conjunction with the expression of acetyl-CoA carboxylase (ACCα) and fatty acid synthase (FAS). Moreover, mature adipocytes further synthesize triglycerides, which

aggregate to form lipid droplets [17]. In addition, the triglycerides in lipid droplets are degraded in conjunction with the expression of adipose triglyceride lipase (ATGL), hormone-sensitive lipase (HSL), and lipoprotein lipase (LPL) [18,19], which are regulated by the AMP-activated protein kinase α (AMPK α) signalling pathway; thermogenesis in BAT is also regulated by this pathway [20,21]. Based on these results, finding an effective agent to regulate metabolic disorders and alleviate diabetes-associated obesity is very important.

Some antiobesity drugs, such as orlistat and liraglutide, have been applied clinically in recent years [22,23]. Orlistat controls body weight by inhibiting pancreatic lipases but has side effects, such as faecal incontinence and flatulence. [24]. Liraglutide controls body weight by suppressing gastric emptying and food intake, increasing satiety, and limiting nutrient absorption by increasing pancreatic β cell proliferation, regenerating β cells, and alleviating insulin resistance but also has side effects such as nausea, vomiting, and diarrhoea [25]. Metformin, a first-line therapeutic agent for diabetes, is an AMPK activator capable of increasing insulin sensitivity and decreasing body weight, but it also has side effects such as abdominal distension, diarrhoea and gastrointestinal intolerance [26]. In recent years, some natural medicinal plants have been used in the treatment of obesity and metabolic diseases because of their safety [27]. *Hibiscus rosa-sinensis* flowers were reported to be capable of decreasing obesity by reducing adipogenesis and activating AMPK to promote fatty acid oxidation [12]. *Momordica charantia* extracts can activate the AMPK signalling pathway, reduce adipogenic gene expression and peroxisome proliferator-activated receptor (PPAR)

signalling in adipose tissue, and increase lipid oxidation in adipose tissue, thereby reducing obesity and insulin resistance [28,29]. In addition, cannabidiol can promote adipocyte browning for the treatment of metabolic diseases [30].

Previously, Teng et al. extracted a proteoglycan called *FYGL* (Fudan-Yueyang *G. lucidum*) from the fruiting body of *Ganoderma lucidum*, a traditional Chinese medicinal herb used for immunoregulation, anti-inflammation, anti-diabetes and anti-cancer[31,32]. The dominant sequence of *FYGL* is shown in Figure 1 [33,34]. *FYGL* is a hyperbranched proteoglycan with a molecular weight of 2.6×10^5 Da and a saccharide: protein ratio of 77:17 [33,34]. *FYGL* has been proven capable of decreasing fasting blood glucose through inhibition of the activity of protein tyrosine phosphatase 1 B (PTP1B), an insulin resistance receptor, both *in vitro* [35] and *in vivo* [36,37], as well as reducing body weight in ob/ob mice [38]. However, the underlying mechanism by which *FYGL* controls body weight is unknown.

In this work, the mechanism of *FYGL* antidiabetic associated with obesity was investigated both *in vivo* and *in vitro*. In *in vivo* studies, adipose tissue from db/db diabetic mice was used to analyse the expression of genes related to fatty acid biosynthesis and metabolism, thermogenesis, and insulin sensitivity, which are beneficial for BAT functions. In *in vitro* studies, the 3T3-L1 cell line was used to investigate the underlying mechanism by which *FYGL* alleviates obesity. 3T3-L1 cells are preadipocytes and normally differentiate into mature adipocytes [39]. The effects of *FYGL* on preadipocyte differentiation and mature adipocyte lipid metabolism were investigated by multiple approaches, including analysis of protein expression in

preadipocytes and the signalling pathways of lipid metabolism in mature adipocytes.

Materials and Methods

Materials

Fruiting bodies of *G. lucidum* grown in northeastern China were purchased from Leiyunshang Pharmaceutical Co. Ltd (Shanghai, China). The preparation of *FYGL* was described in previous work [36]. Dulbecco's modified Eagle's medium (DMEM), foetal bovine serum (FBS), and penicillin/streptomycin antibiotics were purchased from Gibco Co. Ltd (USA). 3T3-L1 cells were obtained from Procell Life Science & Technology Co. Ltd (Wuhan, China). Fluorescein isothiocyanate (FITC), 4′,6-diamidino-2-phenylindole (DAPI), rhodamine-labelled phalloidin and super ECL detection reagent were provided by Yeasen Co. Ltd (Shanghai, China). A cell counting kit-8 (CCK-8), a modified oil red O staining kit, a bicinchoninic acid (BCA) kit, newborn calf serum (NCS), RIPA lysis buffer, dexamethasone, 3-Isobutyl-1-methylxanthine (IBMX), paraformaldehyde, Triton X-100, anti-rabbit IgG (H + L), and a horseradish peroxidase (HRP)-labelled secondary antibody were purchased from Beyotime Co. Ltd (Shanghai, China). Dimethyl sulfoxide (DMSO) was provided by Sigma–Aldrich (Taufkirchen, Germany). Triglyceride (TG) assay kits were obtained from Jiancheng Bioengineering Institute (Nanjing, China). The RNAprep pure cell kit was acquired from TIANGEN Biotech Co. Ltd (Beijing, China). The HiScript III All-in-one RT SuperMix kit (#R333) and Taq Pro Universal SYBR qPCR Master Mix kit (#Q712) were purchased from Vazyme Biotech Co. Ltd (Nanjing, China). Primary antibodies against peroxisome proliferator-activated receptor gamma (PPAR, A11183),

lipoprotein lipase (LPL, A16252), and β-actin (AC026) were purchased from ABclonal Technology Co. Ltd (Wuhan, China). Primary antibodies against CCAAT/enhancer-binding protein α (C/EBPα, ab40764), fatty acid synthase (FAS, ab128870), fatty acid binding protein 4 (FABP-4, ab92501), adipose triglyceride lipase (ATGL, ab109251), AMPKα1 (ab32047), AMPKα1 (phospho T183) + AMPKα2 (phospho T172) (p-AMPKα, ab133448) were purchased from Abcam (Cambridge, MA, USA). Primary antibodies against hormone-sensitive lipase (HSL, #4107) were purchased from Cell Signaling Technology (CST, Beverly, MA, USA).

Animal trial

All male BKS-DB (*db*/*db*) mice (4 weeks old) and wild-type BKS-DB (db/m) mice were purchased from GemPharmatech Co. Ltd, Nanjing, China. Mice were housed in the specific pathogen-free (SPF) Animal Experimental Center of the School of Pharmacy, Fudan University, at a constant temperature (22 ± 2 °C) on a 12 h/12 h light/dark cycle and were provided standard food and water. All animal trials were conducted following protocols approved by the Fudan University Institutional Animal Care and Use Committee. Subsequent experimental procedures were performed according to the method described in previous works [40,41]. Mice were randomly divided into six groups (n = 12 mice per group): (1) normal group (wild-type BKS mice treated with saline); (2) control group (*db*/*db* mice treated with saline); (3) positive control group (*db*/*db* mice treated with 225 mg/kg metformin); (4) low-dose group (*db*/*db* mice treated with 225 mg/kg *FYGL*); (5) middle-dose group (*db*/*db* mice treated with 450 mg/kg *FYGL*); and (6) high-dose group (*db*/*db* mice treated with 900 mg/kg

FYGL). After 7 weeks of drug treatment, all the mice were sacrificed.

Histopathological analysis of beige adipose tissue

Beige adipose tissue (BAT) was extracted from the scapulae of db/db mice and were fixed, sectioned, and mounted. The sections were stained with haematoxylin and eosin (H&E) and observed by microscopy (NanoZoomer 2.0-HT, Japan). Adipocyte numbers are shown as ratios of the adipocyte number to the area of the selected region (a randomly selected circle with an area of 0.1 mm^2) in the images.

RNA sequencing (RNA-seq) analysis of BAT

Total RNA was extracted from beige adipose tissue. RNA purity was checked using a NanoPhotometer® spectrophotometer (IMPLEN, CA, USA). RNA integrity was assessed using the RNA Nano 6000 Assay Kit for the Bioanalyzer 2100 system (Agilent Technologies, CA, USA). Sequencing libraries were generated using the NEBNext® UltraTM RNA Library Prep Kit for Illumina® (NEB, USA) following the manufacturer's recommendations, and index codes were added to attribute sequences to each sample. Clustering of the index-coded samples was performed on a cBot Cluster Generation System using TruSeq PE Cluster Kit v3-cBot-HS (Illumina) according to the manufacturer's instructions. After cluster generation, the library preparations were sequenced on the Illumina NovaSeq platform, and 150 bp paired-end reads were generated. Every group was analysed with three biological replicates. Differential expression analyses between two conditions or groups (two biological replicates per condition) were performed using the DESeq2 R package (1.16.1). Genes with an adjusted P value of < 0.05 determined by DESeq2 were considered differentially

expressed. Gene Ontology (GO) enrichment analysis of the differentially expressed genes was implemented in the cluster Profiler R package, in which gene length bias was corrected. GO terms with a corrected *P* value of less than 0.05 were considered significantly enriched with differentially expressed genes. The clusterProfiler R package was used to test the statistical enrichment of the differentially expressed genes in KEGG pathways.

Cell culture and treatment

3T3-L1 preadipocytes were maintained in DMEM supplemented with 10% NCS and 1% penicillin–streptomycin (basal medium I, BMI). When the cells were confluent (Day 0), adipocyte differentiation was induced by treatment with a cocktail of 5 µg/mL insulin, 1 µM dexamethasone, and 0.5 mM isobutyl methylxanthine in DMEM supplemented with 10% FBS and 1% penicillin–streptomycin (differentiation medium I, DMI). After 48 h (Day 2), the medium was changed to DMEM containing 10% FBS, 1% penicillin–streptomycin, and 5 µg/mL insulin for 48 h (differentiation medium II, DMII). On Day 4, insulin was removed from the medium, and the cells were maintained in DMEM supplemented with 10% FBS and 1% penicillin–streptomycin (basal medium II, BMII), and the medium was changed every two days thereafter [42]. During differentiation, cells were treated with different concentrations of *FYGL* (0, 50, 100, 200, 400, and 800 µg/mL). Undifferentiated cells cultured in BMI were used as the blank control group, and differentiated cells cultured in BMI without *FYGL* were used as the model groups.

Uptake of *FYGL* in 3T3-L1 cells

Three milligrams of FITC fluorescence agent was dissolved in 0.3 mL of DMSO to prepare a FITC solution with a concentration of 10 mg/mL, and then the solution was diluted to 1 mg/mL with sodium buffer (SB). *FYGL* (10 mg) was dissolved in 10 mL SB to form a 1 mg/mL *FYGL* solution, which was mixed with the diluted FITC solution at a volume ratio of 10:1. The mixture was stirred at low temperature (ice bath) to allow the formation of fluorescent FITC–*FYGL* complexes. After the coupling reaction was allowed to proceed overnight, the solution was dialyzed with a 1 kDa dialysis bag to filter free FITC and then cryodesiccated.

3T3-L1 cells were seeded on microscope cover glasses in a 24-well plate at a density of 1×10^4 cells per well and were incubated with FITC–*FYGL* complexes (200 μg/mL) for 4 h. Nuclei and F-actin (filamentous actin) in 3T3-L1 cells were stained by DAPI and phalloidin-TRITC (phalloidin-tetramethyl rhodamine), respectively. Cell images were acquired with a $C2^+$ laser scanning confocal microscope (Nikon, Japan). Moreover, 3T3-L1 cells were treated with the indicated concentrations of FITC–*FYGL* (0, 50, 100, 200, 400, 800 μg/mL) for 4 h, and then the fluorescence intensity was determined by flow cytometry (Gallios, Beckman Coulter) to visualize the uptake of *FYGL* in the cells.

Measurement of cell viability

Cell viability was measured by a cell counting kit-8 (CCK-8) assay. In brief, 3T3-L1 cells were plated into 96-well plates at a density of 5×10^3 cells per well and incubated to near confluence. Some cells were incubated in DMI with different concentrations of *FYGL* (0, 100, 200, 400, and 800 μg/mL) for 24 h. After treatment for 24 h, the medium

was discarded, and fresh DMI containing CCK-8 solution was added to the 96-well plates. Approximately 1 h later, a multimode microplate reader (Cytation3, BioTek, U.S.A.) was used to measure the optical density (OD) at 450 nm.

5

Triglyceride quantification

Triglyceride (TG) concentrations were determined using a commercial kit (Jiancheng Bioengineering Institute, China). Briefly, differentiated 3T3-L1 5 cells were washed twice with phosphate-buffered saline (PBS) and harvested by scraping from the culture plate in PBS containing 1% Triton X-100 on Day 6. 5 Cell homogenates were obtained by sonication, and TG concentrations were determined using a commercial kit according to the manufacturer's instructions. Protein concentrations were measured using the bicinchoninic acid (BCA) protein assay kit (Beyotime, China) and used for quantification of proteins in samples.

6

Oil red O staining and quantification

Lipid accumulation in cells was measured by oil red O staining. Differentiated 3T3-L1 cells were subjected to 17 oil red O staining with modified oil red O staining kits (Beyotime, China). Briefly, 4 the cells were washed with phosphate-buffered saline (PBS, pH 7.4) and then fixed with 10% (v/v) paraformaldehyde at room temperature for 10 min. Then, the fixation solution was removed, and the cells were washed twice with PBS. The cells were immersed in washing solution for 20 secs. After the washing solution was discarded, modified oil red O 4 was added and incubated with the cells at room temperature for 20 min. Then, the staining solution was removed, and the cells were washed with washing solution once and PBS twice. Finally, 6 cells stained with oil

red O were examined via a polarizing microscope (DM2500P, Leica, Germany). In addition to this gross evaluation, the dye was dissolved in 60% isopropanol solution, and the absorbance was measured at 510 nm.

RNA extraction and RT–qPCR analysis

Total RNA was isolated from differentiated 3T3-L1 cells using RNAprep pure cell kits (TIANGEN, China) according to the manufacturer's instructions. Conversion of total RNA to single-stranded cDNA was performed using HiScript III All-in-one RT SuperMix Kits (Vazyme, China). The series of primers shown in Table 1 for amplification of β-actin (as an internal reference), C/EBPα, FABP4, ATGL, and LPL were synthesized by Sangon Co. The primers were mixed with the cDNA templates, and qPCR was then performed with a Taq Pro Universal SYBR qPCR Master Mix kit (Vazyme, China) on a qPCR instrument (Bio-Rad, Germany) to amplify the DNA of C/EBPα, FABP4, ATGL, and LPL. The melt curves of the cDNA were analysed to determine the specificity of amplification, and quantification of relative mRNA levels was performed using the $2^{-\Delta\Delta Ct}$ method with normalization to β-actin mRNA.

Protein extraction and immunoblot analysis

Immunoblot analysis was performed according to the method described in a previous report with a minor modification [43]. Differentiated 3T3-L1 cells were lysed in RIPA lysis buffer and centrifuged (12000 × *g*, 10 min, 4 °C). Proteins in the lysates were separated by 10% SDS–PAGE and transferred to polyvinylidene fluoride membranes. Then, the membranes were blocked in TBST/5% nonfat dry milk powder; incubated overnight at 4 °C with antibodies against FABP4, PPARγ, CEBPα, AMPKα, p-AMPKα,

ATGL, HSL, LPL, and β-actin; and incubated with a goat anti-rabbit secondary antibody at room temperature for 1 h. Finally, enhanced chemiluminescence solution (ECL) was used to detect the proteins on the membranes. The luminescence signals were recorded with a Chemiscope3300 mini (Clinx Science Instruments, China). Data were collected from three independent experiments.

Statistical analysis

All data were analysed by SPSS 20.0 (SPSS, Inc., U.S. and are expressed as the mean ± S.D. values. One-way ANOVA followed by the Bonferroni correction was performed to analyse the statistical significance of differences among the groups. A value of $P < 0.05$ was considered statistically significant.

Results and Discussion

Effect of *FYGL* on BAT histopathology *in vivo*

Teng previously proved that *FYGL* can decrease triglycerides and total cholesterol in SD rats with STZ-induced diabetes [33], which is closely related to lipid biosynthesis and metabolism. In the present work, BAT in db/db mice was subjected to histopathological analysis. Figure 2A shows that the size of beige adipocytes was larger and the numbers were lower in the control group than in the normal group, whereas treatment with metformin and *FYGL* reduced the size of adipocytes. Semiquantitative analysis of H&E staining in Figure 2B showed that *FYGL* significantly increased the number of adipocytes per unit area of BAT in a dose-dependent manner and even outperformed metformin.

Cypess et al. proved that the amount and activity of BAT are inversely correlated

with body mass index [44]; the smaller the size and the greater the number of beige adipocytes, the healthier the body [45-47]. Ouellet et al. demonstrated that the activity of beige adipocytes is positively correlated with the level of glucose uptake in cells, which modulates the blood glucose content [48]. Therefore, increasing the number or activation of beige adipocytes could be a potential approach to treat type 2 diabetes-associated obesity [49]. Consistent with those studies, the results of this study showed that beige adipocytes were significantly enlarged and increased in number in db/db mice, while these changes were significantly reversed after *FYGL* treatment.

Effect of *FYGL* on lipid metabolism *in vivo*

Type 2 diabetes is strongly associated with genes of lipid metabolism[50]. In this work, BAT transcriptome sequencing was performed to explore the potential molecular mechanism of lipid metabolism *in vivo*. As shown in Figure 3A, the screening results of the differentially expressed genes (DEGs) showed that the ratio of upregulated: downregulated: all significant differentially expressed genes was approximately 0.5:0.5:1 in the metformin and *FYGL* groups compared with the control group, nearly the same as the ratio in the normal group compared to the control group. Figure 3B shows the hierarchical clustering heatmap. The large coloured square patterns represent the upregulated or downregulated genes in the different groups. The change in colour from blue to red indicates a change in the gene expression from downregulation to upregulation. The narrow columns on the left show the pathway-related genes. Figure 3B shows that the colour patterns of the DEGs in the control group were different from those in the normal group for most genes except *Ppp1r3b*, while the colour patterns in

the *FYGL* group were similar to those in the normal group. From the pathway indication in the upper-left corner in Figure 3B, it can be seen that the DEGs were involved in the pathways of fatty acid synthesis (black), fatty acid oxidation (green), insulin resistance (yellow), and thermogenesis (purple).

As shown in Figure 3B, *FYGL* increased the mRNA levels of *Ppp1r3b*, *Fasn*, *Acaca*, *CPT2*, and *Acadl* in the BAT of db/db mice compared to those in the control group. The *Ppp1r3b* gene encodes protein phosphatase 1, which is a critical protein in glycogen metabolism regulated by insulin [51]. The *Fasn* (encoding FAS [52]) and *Acaca* (encoding ACCα [53]) genes are involved in fatty acid synthesis [52,53]. *CPT2* (encoding CPT-II, carnitine palmitoyl transferase II [54]) and *Acadl* (encoding ACADL, acyl-CoA dehydrogenase long chain [55]) are involved in the β-oxidation of long-chain fatty acids in mitochondria [54,55]. The imbalance between fatty acid synthesis and degradation can lead to dyslipidaemia, diabetes and cardiovascular disease [56, 57]. Transcript analysis of those genes in BAT indicated that *FYGL* could upregulate fatty acid metabolism *in vivo*. Additionally, as shown in Figure 3B, *FYGL* upregulated fatty acid degradation genes (*CPT2* and *Acadl*) more significantly than fatty acid synthesis genes (*Fasn* and *Acaca*). In addition, as shown in Figure 3C, *FYGL* increased the levels of *Cd81* (encoding CD81 [58]) and *Slc25a4* (encoding SLC25A4 [59]) compared to those in the control group, and the levels of these mRNAs in the *FYGL* group were even higher than those in the metformin group. CD81 is a marker of beige adipocyte progenitors. The absence of CD81 leads to diet-induced obesity, insulin resistance, and adipose tissue inflammation [58]. SLC25A4, a mitochondrial ATP/ADP transporter,

regulates BAT thermogenesis through UCP1-independent mechanisms [60]. Beige adipocytes can produce heat by metabolizing fatty acids. Transcriptome analysis indicated that *FYGL* could increase the expression of thermogenesis genes (*Cd81* and *Slc25a4*) in BAT, as indicated by the transition from blue to red in Figure 3C. Furthermore, *FYGL* and metformin increased the mRNA levels of *Akt2* (encoding AKT2 [61]) and *Slc2a4* (encoding GLUT-4, glucose transporter-4 [62]), as shown in Figure 3B. Deficiency of AKT2 and GLUT-4 leads to type 2 diabetes and insulin resistance [61,63].

The GO (Gene Ontology) database is a comprehensive database describing gene functions and includes the biological process (BP), cellular component (CC), and molecular function (MF) ontological categories. Figure 4A shows the bubble plot of the biological processes in the GO enrichment analysis (*FYGL vs.* control), where the redder the dot is, the more significant the enrichment of the biological process. Figure 4A shows that DEGs were mainly enriched in terms related to the biological processes of cellular respiration, fatty acid metabolism, tricarboxylic acid metabolism, fatty acid oxidation, etc., and that *FYGL* restored BAT functions in db/db mice through those biological processes. Figure 4B is a directed acyclic graph (DAG, *FYGL vs.* control) of the GO biological process enrichment analysis results and indicates the relationship of functions from upregulated to downregulated biological processes. Figure 4C shows that the biological processes were eventually refined to include only fatty acid metabolism and cellular respiration. *FYGL* upregulated the fatty acid metabolism process and promoted thermogenesis in brown adipocytes.

KEGG enrichment analysis aims to identify connections between differentially expressed genes and signalling pathways. Figure 4C shows the bubble plot of the signalling pathways in the KEGG enrichment analysis (*FYGL vs.* control), where the redder the dot is, the more significant the enrichment of the signalling pathway. Figure 4B shows that DEGs were predominantly enriched in signalling pathways related to oxidative phosphorylation, thermogenesis, the citrate cycle (TCA cycle), fatty acid metabolism, and fatty acid biosynthesis. The data in Figure 4B suggest that *FYGL* promotes the functions of BAT through those signalling pathways.

These findings indicated that *FYGL* could balance fatty acid biosynthesis and metabolism to effectively dissipate energy, therefore reducing insulin resistance and increasing insulin sensitivity *in vivo*.

Cellular uptake of *FYGL*

To reveal the underlying mechanisms of *FYGL* in mediating biological functions, investigations at the cellular level are necessary. Figure 5A shows the uptake of *FYGL* (200 μg/mL and 400 μg/mL) in 3T3-L1 cells, as measured by confocal laser scanning microscopy, where green fluorescence was found in the cells cultured with FITC-*FYGL*, indicating that *FYGL* could be taken up well into 3T3-L1 cells. Moreover, the results of flow cytometric analysis of *FYGL* uptake in 3T3-L1 cells are shown in Figure 5B and Figure 5C; the peak of the curve shifted to the right as the FITC–*FYGL* concentration increased, and the uptake of *FYGL* in cells occurred in a dose-dependent manner.

Effect of *FYGL* on cell viability

To examine the cytotoxicity of *FYGL* in 3T3-L1 adipocytes, cell viability was measured using the CCK-8 assay. Adipocytes were treated with various concentrations of *FYGL* (0-800 μg/mL). The CCK-8 assay results shown in Figure 5D demonstrate that *FYGL* had no obvious cytotoxicity at concentrations up to 800 μg/mL.

Effect of *FYGL* on the accumulation of intracellular triglycerides and lipids

Lipid accumulation in adipocytes is a hallmark of adipogenesis. Mature differentiated cells accumulate triglycerides, which then converge to form lipid droplets (LDs). *FYGL* significantly decreased the triglyceride content, as shown in Figure 6A. Moreover, cell differentiation and lipid accumulation can be identified by oil red O staining and triglyceride assays. Figure 6B shows that the number of lipid droplets (red staining) was markedly increased in cells cultured in differentiation medium (DM) but was significantly decreased when the cells were cultured with *FYGL* (200-400 μg/mL), and Figure 6C quantitatively shows the effect of *FYGL* on lipid droplet accumulation. Excessive accumulation of lipid droplets in adipocytes leads to obesity and insulin resistance [17]. *FYGL* inhibited triglyceride accumulation and lipid droplets in differentiated adipocytes. The mechanism of inhibition was further investigated as follows.

Effect of *FYGL* on the expression of adipogenic and lipolytic genes and proteins

Several reports have shown that peroxisome proliferator-activated receptor γ (PPARγ) and CCAAT/enhancer-binding protein α (C/EBPα) are marker proteins of adipocyte differentiation and adipogenesis [64-66]. Tali et al. found that fatty acid binding protein-4 (FABP-4)-null preadipocytes can enhance PPARγ expression and activity,

while the overexpression of FABP-4 inhibits PPARγ expression and adipogenesis [67]. Furuhashi et al. further found that FABP-4-null mice exhibit decreased lipolysis in adipocytes and pancreatic β cells and reduced insulin secretion [68]. To reveal the mechanisms by which *FYGL* inhibits the accumulation of intracellular triglycerides and lipids, the effect of *FYGL* on the expression of adipogenic and lipolytic genes and proteins was investigated. Interestingly, *FYGL* significantly increased the transcript level of FABP-4 (Figure 7A) in 3T3-L1 preadipocytes and considerably increased the transcript level of C/EBPα in adipocytes cultured in differentiation medium (Figure 7B). Moreover, *FYGL* increased the mRNA level of lipolytic genes, such as ATGL (Figure 7C) and LPL (Figure 7D).

Furthermore, Western blotting was used to analyse the protein expression of FABP-4, PPARγ, and C/EBPα, as shown in Figure 8A. *FYGL* greatly increased FABP-4 expression, as shown in Figure 8B, and markedly decreased PPARγ and C/EBPα expression, as shown in Figure 8C and 8D. This work proved that *FYGL* could inhibit the differentiation of 3T3-L1 preadipocytes and promote lipolysis in adipocytes, therefore reducing lipid droplet accumulation.

Effect of *FYGL* on lipid metabolism and the AMPKα signalling pathway

Studies have shown that fatty acid synthase (FAS) plays an important role in lipogenic pathways, which are involved in fatty acid biosynthesis [69]. In addition, the AMPKα signalling pathway also plays a critical role in lipolysis [20,70,71]. Activating the AMPK signalling pathway can increase the activity of the lipases ATGL, HSL, and LPL, thus promoting the utilization of lipid storage [20,70-72]. ATGL and HSL catalyse

triglyceride degradation, releasing the fatty acids in lipid droplets of adipocytes [73], while adipocytes secrete LPL to degrade triglycerides in VLDL in vessels [74]. As shown in Figure 9A and 9B, *FYGL* increased the protein expression of FAS. Additionally, *FYGL* increased the phosphorylation of AMPKα (Figure 9C&D) and consequently increased the protein expression of lipolysis markers, such as ATGL (Figure 9E), HSL (Figure 9F), and LPL (Figure 9G).

The results of this study indicated that *FYGL* promoted the degradation of lipid droplets in mature adipocytes by activating the AMPKα signalling pathway. In addition, *FYGL* increased the protein levels of ATGL (Figure 9E) and HSL (Figure 9F) by 2-fold compared with that of FAS (Figure 9B) and by 1.5-fold compared with those in the control group at concentrations higher than 200 μg/mL. Therefore, *FYGL* upregulated lipolysis more significantly than fatty acid biosynthesis, consistent with the animal experiment results. Taken together, the results of the study on the cellular level showed that *FYGL* could inhibit lipid accumulation by both suppressing the differentiation of preadipocytes and promoting the degradation of lipid droplets in mature adipocytes to alleviate metabolically unhealthy obesity.

Conclusion

In conclusion, this study showed that *FYGL* could increase the number of beige adipocytes and restore adipocyte morphology, thereby alleviating metabolic disorders in db/db mice. The mechanism by which *FYGL* alleviates metabolic disorders involves the balance between fatty acid biosynthesis and metabolism to effectively dissipate energy in beige adipocytes. In addition, *FYGL* inhibited the differentiation of

preadipocytes by increasing FABP-4 gene expression and decreasing PPARγ and C/EBPα gene levels. Moreover, *FYGL* promoted adipocyte browning by upregulating *Cd81* gene expression. Furthermore, *FYGL* increased the levels of the lipolysis-related proteins ATGL, HSL and LPL by activating the AMPKα signalling pathway, therefore accelerating lipid metabolism in mature adipocytes. Importantly, these findings proved that *FYGL*, a proteoglycan, could improve metabolic disorders *in vivo* by targeting both preadipocytes and mature adipocytes. The mechanistic profile of *FYGL* in the treatment of diabetes-associated obesity is shown in Figure 10. *FYGL* could be used as a promising agent to treat lipid metabolism disorders and obesity in the clinic.

Declarations

Ethics approval and consent to participate: The study was conducted in accordance with the Declaration of Helsinki and approved by the Ethics Committee of Fudan University (No. FE21038, 5 March 2021).

Consent for publication: Not applicable.

Availability of data and materials: The data presented in this study are available within the article.

Competing interests: The authors declare that they have no competing interests.

Funding: This research was funded by the National Natural Science Foundation of China (Nos. 21374022 and 81374032); National Health Commission of the People's Republic of China (No. 2017ZX09301006); Science and Technology Commission of Shanghai Municipality (No. 17401902700); Clinical Research Plan of SHDC (No. SHDC12019124); and Shanghai Collaborative Innovation Center of Industrial Transformation of Hospital TCM Preparation.

Authors' contributions: Y.W: design, acquisition, analysis and interpretation of data, writing of the draft and final manuscript version. F.Y: interpretation of data, revision of the draft. X.Z: revision of the draft. J.L: analysis of data. Z.Z: interpretation of data. Q.Z: revision of the draft. J.C: analysis of data. Y.H: interpretation of data, funding support. H.Y: design of the work, funding support. P.Z: design of the work, funding support, writing, reviewing and editing of the manuscript. All authors read and approved the final manuscript.

Acknowledgements: Not applicable.

References

1. Chatterjee S, Khunti K, Davies MJ: **Type 2 diabetes.** *The Lancet* 2017, **389:**2239-2251.
2. Tong Y, Xu S, Huang L, Chen C: **Obesity and insulin resistance: Pathophysiology and treatment.** *Drug Discovery Today* 2022, **27:**822-830.
3. Nathan DM: **Diabetes Advances in Diagnosis and Treatment.** *Jama-Journal of the American Medical Association* 2015, **314:**1052-1062.
4. Dhurandhar NV: **What is obesity?** *International Journal of Obesity* 2022, **46:**1081-1082.
5. Czech MP: **Mechanisms of insulin resistance related to white, beige, and brown adipocytes.** *Molecular Metabolism* 2020, **34:**27-42.
6. Gustafson B, Hedjazifar S, Gogg S, Hammarstedt A, Smith U: **Insulin resistance and impaired adipogenesis.** *Trends in Endocrinology & Metabolism* 2015, **26:**193-200.
7. Dhurandhar NV: **What is obesity?** *International Journal of Obesity* 2022.
8. Romao JM, Guan LL: **Chapter 21 - Adipogenesis and Obesity.** In *MicroRNA in Regenerative Medicine.* Edited by Sen CK. Oxford: Academic Press; 2015: 539-565
9. Hansen Jacob B, Kristiansen K: **Regulatory circuits controlling white versus brown adipocyte differentiation.** *Biochemical Journal* 2006, **398:**153-168.
10. Yang FT, Stanford KI: **Batokines: Mediators of Inter-Tissue Communication (a Mini-Review).** *Current Obesity Reports* 2022, **11:**1-9.
11. Wu M, Liu D, Zeng R, Xian T, Lu Y, Zeng G, Sun Z, Huang B, Huang Q: **Epigallocatechin-3-gallate inhibits adipogenesis through down-regulation of PPARγ and FAS expression mediated by PI3K-AKT signaling in 3T3-L1 cells.** *European Journal of Pharmacology* 2017, **795:**134-142.
12. Lingesh A, Paul D, Naidu VGM, Satheeshkumar N: **AMPK activating and anti adipogenic potential of Hibiscus rosa sinensis flower in 3T3-L1 cells.** *Journal of Ethnopharmacology* 2019, **233:**123-130.
13. Otto TC, Lane MD: **Adipose development: from stem cell to adipocyte.** *Crit Rev Biochem Mol Biol* 2005, **40:**229-242.
14. Tang QQ, Lane MD: **Adipogenesis: from stem cell to adipocyte.** *Annu Rev Biochem* 2012, **81:**715-736.
15. Cristancho AG, Lazar MA: **Forming functional fat: a growing understanding of adipocyte differentiation.** *Nature Reviews Molecular Cell Biology* 2011, **12:**722-734.
16. Lehrke M, Lazar MA: **The Many Faces of PPARγ.** *Cell* 2005, **123:**993-999.
17. Sun Z, Gong J, Wu L, Li P: **Chapter 14 - Imaging Lipid Droplet Fusion and Growth.** In *Methods in Cell Biology. Volume* 116. Edited by Yang H, Li P: Academic Press; 2013: 253-268
18. Bosch M, Parton RG, Pol A: **Lipid droplets, bioenergetic fluxes, and metabolic flexibility.** *Seminars in Cell & Developmental Biology* 2020, **108:**33-46.
19. Klemm RW, Ikonen E: **The cell biology of lipid droplets: More than just a phase.** *Seminars in Cell & Developmental Biology* 2020, **108:**1-3.
20. Herzig S, Shaw RJ: **AMPK: guardian of metabolism and mitochondrial homeostasis.** *Nature Reviews Molecular Cell Biology* 2018, **19:**121-135.
21. López M: **EJE PRIZE 2017: Hypothalamic AMPK: a golden target against obesity?** *European Journal of Endocrinology* 2017, **176:**R235-R246.
22. Aaseth J, Ellefsen S, Alehagen U, Sundfør TM, Alexander J: **Diets and drugs for weight loss and health in obesity – An update.** *Biomedicine & Pharmacotherapy* 2021, **140:**111789.

23. Lingvay I, Sumithran P, Cohen RV, Le Roux CW: **Obesity management as a primary treatment goal for type 2 diabetes: time to reframe the conversation.** *The Lancet* 2021.

24. Liu TT, Liu XT, Chen QX, Shi Y: **Lipase Inhibitors for Obesity: A Review.** *Biomedicine and Pharmacotherapy* 2020, **128**.

25. Drucker DJ: **Mechanisms of Action and Therapeutic Application of Glucagon-like Peptide-1.** *Cell Metab* 2018, **27:**740-756.

26. Foretz M, Guigas B, Viollet B: **Understanding the glucoregulatory mechanisms of metformin in type 2 diabetes mellitus.** *Nature Reviews Endocrinology* 2019, **15:**569-589.

27. Ali Esmail A-S, Muayad Hussein A, Kareema Helal S: **Medicinal plants for the treatment of obesity and overweight: A review.** *World Journal of Biology Pharmacy and Health Sciences* 2022, **10:**001-010.

28. Huang HL, Hong YW, Wong YH, Chen YN, Chyuan JH, Huang CJ, Chao PM: **Bitter melon (Momordica charantia L.) inhibits adipocyte hypertrophy and down regulates lipogenic gene expression in adipose tissue of diet-induced obese rats.** *Br J Nutr* 2008, **99:**230-239.

29. Ramachandra Shobha C, Prashant V, Akila P, Chandini R, Nataraj Suma M, Basavanagowdappa H: **Fifty Percent Ethanolic Extract of Momordica charantia Inhibits Adipogenesis and Promotes Adipolysis in 3T3-L1 Pre-Adipocyte Cells.** *Rep Biochem Mol Biol* 2017, **6:**22-32.

30. Parray HA, Yun JW: **Cannabidiol promotes browning in 3T3-L1 adipocytes.** *Molecular and Cellular Biochemistry* 2016, **416:**131-139.

31. Subhasree PDBaRS: **The Sacred Mushroom "Reishi"-A Review.** *American-Eurasian Journal of Botany* 2008, **1 (3): 107-110, 200**.

32. Bishop KS, Kao CHJ, Xu Y, Glucina MP, Paterson RRM, Ferguson LR: **From 2000years of Ganoderma lucidum to recent developments in nutraceuticals.** *Phytochemistry* 2015, **114:**56-65.

33. Teng BS, Wang CD, Zhang D, Wu JS, Pan D, Pan LF, Yang HJ, Zhou P: **Hypoglycemic effect and mechanism of a proteoglycan from ganoderma lucidum on streptozotocin-induced type 2 diabetic rats.** *Eur Rev Med Pharmacol Sci* 2012, **16:**166-175.

34. Pan D, Wang L, Chen C, Hu B, Zhou P: **Isolation and characterization of a hyperbranched proteoglycan from Ganoderma Lucidum for anti-diabetes.** *Carbohydrate Polymers* 2015, **117:**106-114.

35. Yu F, Wang Y, Teng Y, Yang S, He Y, Zhang Z, Yang H, Ding C-F, Zhou P: **Interaction and Inhibition of a Ganoderma lucidum Proteoglycan on PTP1B Activity for Anti-diabetes.** *ACS Omega* 2021, **6:**29804-29813.

36. Teng B-S, Wang C-D, Yang H-J, Wu J-S, Zhang D, Zheng M, Fan Z-H, Pan D, Zhou P: **A Protein Tyrosine Phosphatase 1B Activity Inhibitor from the Fruiting Bodies of Ganoderma lucidum (Fr.) Karst and Its Hypoglycemic Potency on Streptozotocin-Induced Type 2 Diabetic Mice.** *Journal of Agricultural and Food Chemistry* 2011, **59:**6492-6500.

37. Yang Z, Wu F, He Y, Zhang Q, Zhang Y, Zhou G, Yang H, Zhou P: **A novel PTP1B inhibitor extracted fromGanoderma lucidumameliorates insulin resistance by regulating IRS1-GLUT4 cascades in the insulin signaling pathway.** *Food & Function* 2018, **9:**397-406.

38. Yang Z, Zhang Z, Zhao J, He Y, Yang H, Zhou P: **Modulation of energy metabolism and mitochondrial biogenesis by a novel proteoglycan fromGanoderma lucidum.** *RSC Advances* 2019, **9:**2591-2598.

39. Vohra MS, Ahmad B, Serpell CJ, Parhar IS, Wong EH: **Murine in vitro cellular models to**

better understand adipogenesis and its potential applications. *Differentiation* 2020, **115:**62-84.

40. Zhang Y, Pan Y, Li J, Zhang Z, He Y, Yang H, Zhou P: **Inhibition on α-Glucosidase Activity and Non-Enzymatic Glycation by an Anti-Oxidative Proteoglycan from Ganoderma lucidum.** *Molecules* 2022, **27:**1457.
41. Pan Y, Yuan S, Teng Y, Zhang Z, He Y, Zhang Y, Liang H, Wu X, Li J, Yang H, Zhou P: **Antioxidation of a proteoglycan from Ganoderma lucidum protects pancreatic β-cells against oxidative stress-induced apoptosis in vitro and in vivo.** *International Journal of Biological Macromolecules* 2022, **200:**470-486.
42. Zebisch K, Voigt V, Wabitsch M, Brandsch M: **Protocol for effective differentiation of 3T3-L1 cells to adipocytes.** *Analytical Biochemistry* 2012, **425:**88-90.
43. Liang H, Pan Y, Teng Y, Yuan S, Wu X, Yang H, Zhou P: **A proteoglycan extract from Ganoderma Lucidum protects pancreatic beta-cells against STZ-induced apoptosis.** *Bioscience, Biotechnology, and Biochemistry* 2020, **84:**2491-2498.
44. Cypess AM, Lehman S, Williams G, Tal I, Rodman D, Goldfine AB, Kuo FC, Palmer EL, Tseng Y-H, Doria A, .:t al: **Identification and Importance of Brown Adipose Tissue in Adult Humans.** *New England Journal of Medicine* 2009, **360:**1509-1517.
45. Stock MJ, Cinti S: **ADIPOSE TISSUE | Structure and Function of Brown Adipose Tissue.** In *Encyclopedia of Food Sciences and Nutrition (Second Edition).* Edited by Caballero B. Oxford: Academic Press; 2003: 29-34
46. Virtanen KA: **Adipose Tissue: Structure and Function of Brown Adipose Tissue.** In *Encyclopedia of Food and Health.* Edited by Caballero B, Finglas PM, Toldrá F. Oxford: Academic Press; 2016: 30-34
47. Lidell ME, Betz MJ, Leinhard OD, Heglind M, Elander L, Slawik M, Mussack T, Nilsson D, Romu T, Nuutila P, .:t al: **Evidence for two types of brown adipose tissue in humans.** *Nature Medicine* 2013, **19:**631-634.
48. Ouellet V, Routhier-Labadie A, Bellemare W, Lakhal-Chaieb L, Turcotte E, Carpentier AC, Richard D: **Outdoor Temperature, Age, Sex, Body Mass Index, and Diabetic Status Determine the Prevalence, Mass, and Glucose-Uptake Activity of 18F-FDG-Detected BAT in Humans.** *The Journal of Clinical Endocrinology & Metabolism* 2011, **96:**192-199.
49. Kajimura S, Bruce, Seale P: **Brown and Beige Fat: Physiological Roles beyond Heat Generation.** *Cell Metabolism* 2015, **22:**546-559.
50. Kane JP, Pullinger CR, Goldfine ID, Malloy MJ: **Dyslipidemia and diabetes mellitus: Role of lipoprotein species and interrelated pathways of lipid metabolism in diabetes mellitus.** *Current Opinion in Pharmacology* 2021, **61:**21-27.
51. Ceperuelo-Mallafré V, Ejarque M, Serena C, Duran X, Montori-Grau M, Rodríguez MA, Yanes O, Núñez-Roa C, Roche K, Puthanveetil P, .:t al: **Adipose tissue glycogen accumulation is associated with obesity-linked inflammation in humans.** *Molecular Metabolism* 2016, **5:**5-18.
52. Wallace M, Metallo CM: **Tracing insights into de novo lipogenesis in liver and adipose tissues.** *Seminars in Cell & Developmental Biology* 2020, **108:**65-71.
53. Munday MR: **Regulation of mammalian acetyl-CoA carboxylase.** *Biochem Soc Trans* 2002, **30:**1059-1064.
54. Hsiao Y-S, Jogl G, Esser V, Tong L: **Crystal structure of rat carnitine palmitoyltransferase**

II (CPT-II). *Biochemical and Biophysical Research Communications* 2006, **346:**974-980.

55. Chen Y, Ren Q, Zhou Z, Deng L, Hu L, Zhang L, Li Z: **HWL-088, a new potent free fatty acid receptor 1 (FFAR1) agonist, improves glucolipid metabolism and acts additively with metformin in ob/ob diabetic mice.** *Br J Pharmacol* 2020, **177:**2286-2302.
56. Jaiswal M, Schinske A, Pop-Busui R: **Lipids and lipid management in diabetes.** *Best Practice & Research Clinical Endocrinology & Metabolism* 2014, **28:**325-338.
57. Taskinen M-R, Borén J: **New insights into the pathophysiology of dyslipidemia in type 2 diabetes.** *Atherosclerosis* 2015, **239:**483-495.
58. Oguri Y, Shinoda K, Kim H, Alba DL, Bolus WR, Wang Q, Brown Z, Pradhan RN, Tajima K, Yoneshiro T, .:t al: **CD81 Controls Beige Fat Progenitor Cell Growth and Energy Balance via FAK Signaling.** *Cell* 2020, **182:**563-577.e520.
59. Clémençon B, Babot M, Trézéguet V: **The mitochondrial ADP/ATP carrier (SLC25 family): Pathological implications of its dysfunction.** *Molecular Aspects of Medicine* 2013, **34:**485-493.
60. Cohen P, Kajimura S: **The cellular and functional complexity of thermogenic fat.** *Nature Reviews Molecular Cell Biology* 2021, **22:**393-409.
61. Hay N: **Akt isoforms and glucose homeostasis – the leptin connection.** *Trends in Endocrinology & Metabolism* 2011, **22:**66-73.
62. Corrêa-Giannella ML, Machado UF: **SLC2A4gene: a promising target for pharmacogenomics of insulin resistance.** *Pharmacogenomics* 2013, **14:**847-850.
63. Li G, Zhang L: **miR-335-5p aggravates type 2 diabetes by inhibiting SLC2A4 expression.** *Biochemical and Biophysical Research Communications* 2021, **558:**71-78.
64. Rosen ED, Macdougald OA: **Adipocyte differentiation from the inside out.** *Nature Reviews Molecular Cell Biology* 2006, **7:**885-896.
65. Ali AT, Hochfeld WE, Myburgh R, Pepper MS: **Adipocyte and adipogenesis.** *Eur J Cell Biol* 2013, **92:**229-236.
66. Otto TC, Lane MD: **Adipose Development: From Stem Cell to Adipocyte.** *Critical Reviews in Biochemistry and Molecular Biology* 2005, **40:**229 - 242.
67. Garin-Shkolnik T, Rudich A, Hotamisligil GS, Rubinstein M: **FABP4 Attenuates PPARγ and Adipogenesis and Is Inversely Correlated With PPARγ in Adipose Tissues.** *Diabetes* 2014, **63:**900-911.
68. Furuhashi M: **Fatty Acid-Binding Protein 4 in Cardiovascular and Metabolic Diseases.** *Journal of Atherosclerosis and Thrombosis* 2019, **26:**216-232.
69. Berndt J, Kovacs P, Ruschke K, Klöting N, Fasshauer M, Schön MR, Körner A, Stumvoll M, Blüher M: **Fatty acid synthase gene expression in human adipose tissue: association with obesity and type 2 diabetes.** *Diabetologia* 2007, **50:**1472-1480.
70. Grabner GF, Xie H, Schweiger M, Zechner R: **Lipolysis: cellular mechanisms for lipid mobilization from fat stores.** *Nature Metabolism* 2021, **3:**1445-1465.
71. Wang Y, Rodrigues B: **Intrinsic and extrinsic regulation of cardiac lipoprotein lipase following diabetes.** *Biochimica et Biophysica Acta (BBA) - Molecular and Cell Biology of Lipids* 2015, **1851:**163-171.
72. Walther TC, Farese RV: **Lipid Droplets and Cellular Lipid Metabolism.** *Annual Review of Biochemistry* 2012, **81:**687-714.
73. Ahmadian M, Abbott Marcia J, Tang T, Hudak Carolyn SS, Kim Y, Bruss M, Hellerstein Marc K,

Lee H-Y, Samuel Varman T, Shulman Gerald I, .:t al: **Desnutrin/ATGL Is Regulated by AMPK and Is Required for a Brown Adipose Phenotype.** *Cell Metabolism* 2011, **13:**739-748.

74. Borén J, Taskinen M-R, Björnson E, Packard CJ: **Metabolism of triglyceride-rich lipoproteins in health and dyslipidaemia.** *Nature Reviews Cardiology* 2022, **19:**577-592.

658

659

(A)

(B)

Figure 1 (A) The dominant polysaccharide sequence of *FYGL* characterized by chemical analysis and NMR spectroscopy[34]. Rs represents the carbohydrate residues of →2,4)-α-L-Rhap-(1→, →6-β-D-Galp-1→, Araf-(1→ or →3,6)-β-D-Galp-(1→. Protein moieties are covalently bonded with carbohydrate moieties by Ser and Thr residues in the -O- linkage. (B) The dominant sequence of the protein moieties of *FYGL* characterized by mass spectrometry.

(A)

(B)

Figure 2 Histopathological analysis of adipocytes in brown adipose tissues. (A) Representative images of H&E-stained brown adipose tissues, magnification 100×. The scale bar represents 250 μm. (B) Semiquantitative analysis of the adipocyte number per area in BAT by Image-Pro Plus 6.0 software. The mean ± S.D. values are presented (n = 6; $^{***}P < 0.001$ *vs.* normal; $^{\#}P < 0.05$, $^{\#\#}P < 0.01$, $^{\#\#\#}P < 0.001$ *vs.* control).

(A)

(B)

Figure 3 Transcriptome analysis of RNA sequencing of BAT in the normal, metformin, and *FYGL* groups compared to the control group. (A) DEG counts. (B) Hierarchical clustering heatmap of the expression profile of the DEGs.

(A)

(B)

(C)

Figure 4 GO and KEGG functional enrichment analyses based on the DEGs. (A) Bubble plot of biological processes in the GO enrichment analysis. (B) The directed acyclic graph of biological process in the GO enrichment analysis. (C) Bubble plot of pathways in the KEGG enrichment analysis.

Figure 5 (A) Laser confocal scanning microscopy images of *FYGL* in 3T3-L1 cells at 200× magnification. 3T3-L1 cells were incubated with FITC–*FYGL* (200 μg/mL) for 4 h; blue (DAPI labelled), red (rhodamine labelled) and green (FITC labelled) represent the nucleus, cytoskeleton and *FYGL*, respectively. The scale bar represents 100 μm. (B) Flow cytometric analysis of fluorescence. (C) Geometric means calculated by FlowJo software. The data are presented as the mean ± S.D. values (n = 3). $^{***}P < 0.001$ *vs.* control group. (D) Effect of *FYGL* on cell viability. 3T3-L1 cells were incubated with various concentrations of *FYGL* (0, 50, 100, 200, 400 and 800 μg·mL^{-1}) for 24 h, and cell viability was determined by a CCK-8 assay. The mean ± S.D. values are presented (n = 6)

671

(A)

(B)

(C)

Figure 6 Effect of *FYGL* on the inhibition of lipid accumulation in mature adipocytes. Differentiated 3T3-L1 cells were incubated with *FYGL* at concentrations ranging from 0–400 μg/mL. (A) Intracellular TG in mature adipocytes. (B) Intracellular lipid droplets stained by oil red O and visualized by polarized phase contrast microscopy (500×). (C) Intracellular lipid accumulation was quantitatively measured using a microplate reader at an absorbance of 490 nm. Mean ± S.D. values are presented (n = 6). $^{###}P < 0.001$ *vs.* blank control group, $^{**}P < 0.01$, $^{*}P < 0.05$ *vs.* model group.

672

Figure 7 The relative mRNA expression levels of (A) C/EBPα, (B) FABP-4, (C) ATGL, and (D) LPL in differentiated 3T3-L1 cells, with reference to the model group. Data are presented as the mean ± S.D. values (n = 6). $^{\#\#\#}P < 0.001$, $^{\#\#}P < 0.01$, $^{\#}P < 0.05$ *vs.* blank control group. $^{***}P < 0.001$, $^{**}P < 0.01$, $^{*}P < 0.05$ *vs.* model group

Figure 8 Western blot analysis of proteins involved in cellular differentiation in mature 3T3-L1 cells. (A) Images of the PPARγ, C/EBPα, and FABP-4 protein bands relative to the β-actin protein band. (B), (C) and (D) Relative expression of PPARγ, C/EBPα, and FABP-4, respectively, with reference to β-actin, and normalized to the model group. Data are presented as the mean ± S.D. values (n = 3). $^{\#\#}P < 0.01$, $^{\#}P < 0.05$ *vs.* blank control group, $^{***}P < 0.001$, $^{**}P < 0.01$, $^{*}P < 0.05$ *vs.* model group.

678

Figure 9 Western blot analysis of proteins involved in lipolysis and the AMPKα signalling pathway in mature 3T3-L1 cells. (A) Image of FAS protein bands, (B) Quantification of FAS expression. (C) Images of ATGL, HSL, LPL, p-AMPKα, and AMPKα protein bands. (D), (E), (F) and (G) Quantification of ATGL, HSL, LPL, and p-AMPKα/AMPKα protein levels. The protein levels in the model group are normalized to a value of 1.0. Data are presented as the mean ± S.D. values (n = 3). $^{\#\#}P < 0.01$, $^{\#\#\#}P < 0.001$ *vs.* blank control group, $^{**}P < 0.01$, $^{*}P < 0.05$ *vs.* model group.

681

Figure 10 Profile of the mechanism of *FYGL* in ameliorating diabetes-associated obesity

20230405201350841085309659541504

ORIGINALITY REPORT

40%
SIMILARITY INDEX

PRIMARY SOURCES

1	www.mdpi.com<br>Internet	300 words — 5%
2	academic.oup.com<br>Internet	250 words — 4%
3	www.biorxiv.org<br>Internet	133 words — 2%
4	Mengqing Wu, Dan Liu, Rong Zeng, Tao Xian, Yi Lu, Guohua Zeng, Zhangzetian Sun, Bowei Huang, Qiren Huang. "Epigallocatechin-3-gallate inhibits adipogenesis through down-regulation of PPARγ and FAS expression mediated by PI3K-AKT signaling in 3T3-L1 cells", European Journal of Pharmacology, 2017<br>Crossref	87 words — 1%
5	scholarworks.umass.edu<br>Internet	87 words — 1%
6	Shilin Yuan, Yanna Pan, Zeng Zhang, Yanming He, Yilong Teng, Haohui Liang, Xiao Wu, Hongjie Yang, Ping Zhou. "Amelioration of the lipogenesis, oxidative stress and apoptosis of hepatocytes by a novel proteoglycan from <i>Ganoderma lucidum</i>", Biological and Pharmaceutical Bulletin, 2020<br>Crossref	84 words — 1%

7 www.researchgate.net
Internet
69 words — 1%

8 interstellarsuperherbs.com
Internet
67 words — 1%

9 Dong Xu, Tao Qiao, Yue Wang, Qiang-Song Wang, Yuan-Lu Cui. "Alginate nanogels-based thermosensitive hydrogel to improve antidepressant-like effects of albiflorin via intranasal delivery", Drug Delivery, 2021
Crossref
57 words — 1%

10 journals.plos.org
Internet
48 words — 1%

11 Yanna Pan, Shilin Yuan, Yilong Teng, Zeng Zhang et al. "Antioxidation of a proteoglycan from Ganoderma lucidum protects pancreatic β-cells against oxidative stress-induced apoptosis in vitro and in vivo", International Journal of Biological Macromolecules, 2022
Crossref
41 words — 1%

12 pubs.rsc.org
Internet
37 words — 1%

13 www.hindawi.com
Internet
37 words — 1%

14 www.researchsquare.com
Internet
34 words — 1%

15 lipidworld.biomedcentral.com
Internet
30 words — 1%

16 www.frontiersin.org
Internet
30 words — 1%

17 Yang Jiang, Shijie Ding, Feng Li, Chen Zhang, Dongxiao Sun-Waterhouse, Yilun Chen, Dapeng Li. "Effects of (+)-catechin on the differentiation and lipid metabolism of 3T3-L1 adipocytes", Journal of Functional Foods, 2019 — 27 words — < 1%

Crossref

---

18 Shilin Yuan, Yanna Pan, Zeng Zhang, Yanming He, Yilong Teng, Haohui Liang, Xiao Wu, Hongjie Yang, Ping Zhou. "Amelioration of the Lipogenesis, Oxidative Stress and Apoptosis of Hepatocytes by a Novel Proteoglycan from <i>Ganoderma lucidum</i>", Biological and Pharmaceutical Bulletin, 2020 — 23 words — < 1%

Crossref

---

19 www.spandidos-publications.com — 23 words — < 1%

Internet

---

20 Yanna Pan, Ying Zhang, Jiaqi Li, Zeng Zhang, Yanming He, Qingjie Zhao, Hongjie Yang, Ping Zhou. "A proteoglycan isolated from Ganoderma lucidum attenuates diabetic kidney disease by inhibiting oxidative stress-induced renal fibrosis both in vitro and in vivo", Journal of Ethnopharmacology, 2023 — 20 words — < 1%

Crossref

---

21 Ying Zhang, Yanna Pan, Jiaqi Li, Zeng Zhang, Yanming He, Hongjie Yang, Ping Zhou. "Inhibition on α-Glucosidase Activity and Non-Enzymatic Glycation by an Anti-Oxidative Proteoglycan from Ganoderma lucidum", Molecules, 2022 — 20 words — < 1%

Crossref

---

22 Zhou Yang, Fan Wu, Yanming He, Qiang Zhang, Yuan Zhang, Guangrong Zhou, Hongjie Yang, Ping Zhou. " A novel PTP1B inhibitor extracted from ameliorates — 20 words — < 1%

insulin resistance by regulating IRS1-GLUT4 cascades in the insulin signaling pathway ", Food & Function, 2018
Crossref

23	mdpi-res.com<br>Internet	20 words — < 1%
24	Junpei Yamamoto, Takumi Yamane, Yuichi Oishi, Kazuo Kobayashi-Hattori. "Perfluorooctanoic acid binds to peroxisome proliferator-activated receptor y and promotes adipocyte differentiation in 3T3-L1 adipocytes", Bioscience, Biotechnology, and Biochemistry, 2014<br>Crossref	19 words — < 1%
25	Metabolic Syndrome, 2016.<br>Crossref	19 words — < 1%
26	atvb.ahajournals.org<br>Internet	19 words — < 1%
27	insight.jci.org<br>Internet	19 words — < 1%
28	res.mdpi.com<br>Internet	19 words — < 1%
29	Yilong Teng, Haohui Liang, Zeng Zhang, Yanming He, Yanna Pan, Shilin Yuan, Xiao Wu, Qingjie Zhao, Hongjie Yang, Ping Zhou. "Biodistribution and immunomodulatory activities of a proteoglycan isolated from Ganoderma lucidum", Journal of Functional Foods, 2020<br>Crossref	18 words — < 1%
30	aging-us.com<br>Internet	18 words — < 1%

31 Bao, Qinwen, Xiaozhu Shen, Li Qian, Chen Gong, Maoxiao Nie, and Yan Dong. "Anti-diabetic activities of catalpol in db/db mice", Korean Journal of Physiology and Pharmacology, 2016.
Crossref
17 words — < 1%

32 foodandnutritionresearch.net
Internet
17 words — < 1%

33 worldwidescience.org
Internet
17 words — < 1%

34 www.cellandbioscience.com
Internet
17 words — < 1%

35 www.wjgnet.com
Internet
17 words — < 1%

36 "The Lingzhi Mushroom Genome", Springer Science and Business Media LLC, 2021
Crossref
16 words — < 1%

37 Ji Young Je, Jae Eun Park, Youngwan Seo, Ji Sook Han. "HM-chromanone inhibits adipogenesis by regulating adipogenic transcription factors and AMPK in 3T3-L1 adipocytes", European Journal of Pharmacology, 2021
Crossref
16 words — < 1%

38 aac.asm.org
Internet
16 words — < 1%

39 clinicalproteomicsjournal.biomedcentral.com
Internet
16 words — < 1%

40 oacis.repo.nii.ac.jp
Internet
16 words — < 1%

41	Christopher Church, Mallory Brown, Matthew S Rodeheffer. "Conditional immortalization of primary adipocyte precursor cells", Adipocyte, 2015<br>Crossref	15 words — < 1%
42	Jinye Huang, Jun Li, Hui Chen, Chensi Shen, Yuezhong Wen. "Ecological Toxicity Alleviation of Imazethapyr to Non-target Plant Wheat: Active Regulation Between Auxin and DIMBOA", Research Square Platform LLC, 2023<br>Crossref Posted Content	15 words — < 1%
43	Kang Song, Yifan Zhang, Qin Ga, Zhenzhong Bai, Ri-Li Ge. "High-altitude chronic hypoxia ameliorates obesity-induced non-alcoholic fatty liver disease in mice by regulating mitochondrial and AMPK signaling", Life Sciences, 2020<br>Crossref	15 words — < 1%
44	koreascience.kr<br>Internet	15 words — < 1%
45	kclpure.kcl.ac.uk<br>Internet	14 words — < 1%
46	hwmaint.fasebj.org<br>Internet	13 words — < 1%
47	link.springer.com<br>Internet	13 words — < 1%
48	d197for5662m48.cloudfront.net<br>Internet	12 words — < 1%
49	www.intechopen.com<br>Internet	12 words — < 1%

50 Yu-Shan Hsiao, Gerwald Jogl, Victoria Esser, Liang Tong. "Crystal structure of rat carnitine palmitoyltransferase II (CPT-II)", Biochemical and Biophysical Research Communications, 2006 — 11 words — < 1%
Crossref

51 Hao Huang, Tarun Belwal, Li Li, Yanqun Xu, Ligen Zou, Xingyu Lin, Zisheng Luo. "Amphiphilic and Biocompatible DNA Origami ‐ Based Emulsion Formation and Nanopore Release for Anti ‐ Melanogenesis Therapy", Small, 2021 — 10 words — < 1%
Crossref

52 Lau, P., Z. K. Tuong, S.-C. Wang, R. L. Fitzsimmons, J. Goode, G. P. Thomas, G. J. Cowin, M. A. Pearen, K. Mardon, J. L. Stow, and G. E. O. Muscat. "Ror deficiency and decreased adiposity are associated with induction of thermogenic gene expression in subcutaneous white and brown adipose tissue.", AJP Endocrinology and Metabolism, 2014. — 10 words — < 1%
Crossref

53 Menghua Zhang, Xiaoxue Liu, Zhiyao Chen, Shenhao Jiang, Lin Wang, Min Tao, Liyan Miao. "Method development and validation for simultaneous determination of six tyrosine kinase inhibitors and two active metabolites in human plasma/serum using UPLC–MS/MS for therapeutic drug monitoring", Journal of Pharmaceutical and Biomedical Analysis, 2021 — 10 words — < 1%
Crossref

54 bmccomplementmedtherapies.biomedcentral.com — 10 words — < 1%
Internet

diposit.ub.edu

55 Internet

10 words — < 1%

56 shareok.org
Internet

10 words — < 1%

57 www.sciencegate.app
Internet

10 words — < 1%

58 Ae Park, S.. "Genistein and daidzein modulate hepatic glucose and lipid regulating enzyme activities in C57BL/KsJ-db/db mice", Life Sciences, 20060815
Crossref

9 words — < 1%

59 Eva García-Escobar. "Effect of insulin analogues on 3t3-l1 adipogenesis and lipolysis : INSULIN ANALOGUES ON 3T3-L1 ADIPOGENESIS AND LIPOLYSIS", European Journal of Clinical Investigation, 09/2011
Crossref

9 words — < 1%

60 Geng Li, Linghui Zhang. "miR-335-5p aggravates type 2 diabetes by inhibiting SLC2A4 expression", Biochemical and Biophysical Research Communications, 2021
Crossref

9 words — < 1%

61 Jun Shu, Nan Li, Wenshi Wei, Li Zhang. "Detection of molecular signatures and pathways shared by Alzheimer’s disease and type 2 diabetes", Gene, 2021
Crossref

9 words — < 1%

62 Jung Seok HWANG, Su Bi LEE, Mi-Jung CHOI, Jun Tae KIM, Han Geuk SEO. "Anti-adipogenic effect of a turmeric extract-loaded nanoemulsion in 3T3-L1 preadipocytes and high fat diet-fed mice", Food Science and Technology, 2019
Crossref

9 words — < 1%

63 Lei Wang, Chao Li, Qiang Huang, Xiong Fu. " Polysaccharide from Tratt Fruit Attenuates Hyperglycemia and Hyperlipidemia and Regulates Colon Microbiota in Diabetic Mice ", Journal of Agricultural and Food Chemistry, 2019
Crossref
9 words — < 1%

64 Reddy Sankaran Karunakaran, Oruganti Lokanatha, Ganjayi Muni Swamy, Chintha Venkataramaiah et al. "Anti-Obesity and Lipid Lowering Activity of Bauhiniastatin-1 is Mediated Through PPAR-γ/AMPK Expressions in Diet-Induced Obese Rat Model", Frontiers in Pharmacology, 2021
Crossref
9 words — < 1%

65 Yue Tong, Sai Xu, Lili Huang, Chen Chen. "Obesity and insulin resistance: pathophysiology and treatment", Drug Discovery Today, 2021
Crossref
9 words — < 1%

66 bmcpsychiatry.biomedcentral.com
Internet
9 words — < 1%

67 hdl.handle.net
Internet
9 words — < 1%

68 jneuroinflammation.biomedcentral.com
Internet
9 words — < 1%

69 tessera.spandidos-publications.com
Internet
9 words — < 1%

70 www.dovepress.com
Internet
9 words — < 1%

71 www.nature.com
Internet
9 words — < 1%

72 www.scielo.br
Internet
9 words — < 1%

73 www.tesisenred.net
Internet
9 words — < 1%

74 "Cell Biology and Translational Medicine, Volume 2", Springer Science and Business Media LLC, 2018
Crossref
8 words — < 1%

75 Rulifson, I. C., J. Z. Majeti, Y. Xiong, A. Hamburger, K.-J. Lee, L. Miao, M. Lu, J. Gardner, Y. Gong, H. Wu, R. Case, W.-C. Yeh, W. G. Richards, H. Baribault, and Y. Li. "Inhibition of Secreted Frizzled-Related Protein 5 Improves Glucose Metabolism", AJP Endocrinology and Metabolism, 2014.
Crossref
8 words — < 1%

76 Yuli Zhang, Ni Chai, Zhenzhen Wei, Zan Li et al. "YYFZBJS inhibits colorectal tumorigenesis by enhancing Tregs-induced immunosuppression through HIF-1α mediated hypoxia in vivo and in vitro", Phytomedicine, 2022
Crossref
8 words — < 1%

77 Zhou Yang, Congheng Chen, Juan Zhao, Weijie Xu, Yanming He, Hongjie Yang, Ping Zhou. "Hypoglycemic mechanism of a novel proteoglycan, extracted from Ganoderma lucidum , in hepatocytes", European Journal of Pharmacology, 2018
Crossref
8 words — < 1%

78 downloads.hindawi.com
Internet
8 words — < 1%

79 jcancer.org
Internet
8 words — < 1%

jeccr.biomedcentral.com

80 Internet 8 words — < 1%

81 jitc.bmj.com
Internet 8 words — < 1%

82 mdpi.com
Internet 8 words — < 1%

83 nutritionandmetabolism.biomedcentral.com
Internet 8 words — < 1%

84 opendiabetesjournal.com
Internet 8 words — < 1%

85 revistabionatura.com
Internet 8 words — < 1%

86 waseda.repo.nii.ac.jp
Internet 8 words — < 1%

87 www.science.gov
Internet 8 words — < 1%

88 Angiogenesis in Adipose Tissue, 2013.
Crossref 7 words — < 1%

89 Haohui Liang, Yanna Pan, Yilong Teng, Shilin Yuan, Xiao Wu, Hongjie Yang, Ping Zhou. " A proteoglycan extract from protects pancreatic beta-cells against STZ-induced apoptosis ", Bioscience, Biotechnology, and Biochemistry, 2020
Crossref 7 words — < 1%

90 Zhou Yang, Fan Wu, Hongjie Yang, Ping Zhou. "Endocytosis mechanism of a novel proteoglycan, 7 words — < 1%

extracted from Ganoderma lucidum, in HepG2 cells", RSC Adv., 2017
Crossref

---

91 "Mechanisms involved in the loss of brown adipose tissue in the AGPAT2 deficient mouse.", Pontificia Universidad Catolica de Chile, 2019 — 6 words — < 1%
Crossref Posted Content

---

92 "Principles of Diabetes Mellitus", Springer Science and Business Media LLC, 2017 — 6 words — < 1%
Crossref

---

93 Albert Boronat-Toscano, Diandra Monfort-Ferré, Margarita Menacho, Aleidis Caro et al. "Anti-TNF Therapies Suppress Adipose Tissue Inflammation in Crohn's Disease", International Journal of Molecular Sciences, 2022 — 6 words — < 1%
Crossref

---

94 Lijun Chi, Dorothy Lee, Sharon Leung, Guanlan Hu et al. "Peroxisome loss leads to increased mitochondrial biogenesis and reduced autophagy to preserve mitochondrial function", Research Square Platform LLC, 2023 — 6 words — < 1%
Crossref Posted Content

---

95 Medina-GÃ³mez, Gema. "Mitochondria and endocrine function of adipose tissue", Best Practice & Research Clinical Endocrinology & Metabolism, 2012. — 6 words — < 1%
Crossref

---

96 Min, Byulchorong, Heejin Lee, Ji Hye Song, Myung Joo Han, and Jayong Chung. "Arctiin inhibits adipogenesis in 3T3-L1 cells and decreases adiposity and body weight in mice fed a high-fat diet", Nutrition Research and Practice, 2014. — 6 words — < 1%
Crossref

97	Walter Wahli. "Fat poetry: a kingdom for PPARγ", Cell Research, 06/2007<br>Crossref	6 words — < 1%

EXCLUDE QUOTES	OFF	EXCLUDE SOURCES	OFF
EXCLUDE BIBLIOGRAPHY	ON	EXCLUDE MATCHES	OFF
